# RNA sequencing reveals region-specific molecular mechanisms associated with epileptogenesis in a model of classical hippocampal sclerosis

**DOI:** 10.1038/srep22416

**Published:** 2016-03-03

**Authors:** A. S. Vieira, A. H. de Matos, A. M. do Canto, C. S. Rocha, B. S. Carvalho, V. D. B. Pascoal, B. Norwood, S. Bauer, F. Rosenow, R. Gilioli, F. Cendes, I. Lopes-Cendes

**Affiliations:** 1 Department of Medical Genetics, School of Medical Sciences, University of Campinas - UNICAMP, SP, BRAZIL; 2 Faculty of Basic Sciences, Fluminense Federal University, Nova Friburgo, Rio de Janeiro, Brazil UFF; 3 Department of Neurology, Epilepsy Center Hessen, Philipps-University Marburg, Marburg, Germany; 4 Laboratory of Animal Quality Control (CEMIB), Rua 05 de junho, s/n. Cidade Universitária “Zeferino Vaz”. Distrito de Barão Geraldo, Campinas, SP, 13083-877, BRAZIL; 5 Department of Neurology, University of Campinas – UNICAMP, Campinas, SP, Brazil

## Abstract

We report here the first complete transcriptome analysis of the dorsal (dDG) and ventral dentate gyrus (vDG) of a rat epilepsy model presenting a hippocampal lesion with a strict resemblance to classical hippocampal sclerosis (HS). We collected the dDG and vDG by laser microdissection 15 days after electrical stimulation and performed high-throughput RNA-sequencing. There were many differentially regulated genes, some of which were specific to either of the two sub-regions in stimulated animals. Gene ontology analysis indicated an enrichment of inflammation-related processes in both sub-regions and of axonal guidance and calcium signaling processes exclusively in the vDG. There was also a differential regulation of genes encoding molecules involved in synaptic function, neural electrical activity and neuropeptides in stimulated rats. The data presented here suggests, in the time point analyzed, a remarkable interaction among several molecular components which takes place in the damaged hippocampi. Furthermore, even though similar mechanisms may function in different regions of the DG, the molecular components involved seem to be region specific.

Induced, or non-genetic epilepsy animal models, generally rely on the administration of chemoconvulsants or the use of electrical stimulation paradigms^1^,^2^,^3^. Such strategies generally lead to an episode of convulsive *status epilepticus* (SE), induce variable distribution and intensity of hippocampal neuronal loss, and display significant extra-hippocampal damage^4^,^5^,^6^. Animal models of chronic disorders such as mesial temporal lobe epilepsy (MTLE) provide the unique opportunity to observe the establishment and the development of the pathologic process over time, which is not feasible in patients. Therefore, one would prefer to study animal models displaying a phenotype as similar as possible to that of the human condition. Currently, it is well recognized that a significant proportion of patients with MTLE display the classical hallmarks of hippocampal sclerosis (HS), which is characterized by loss of pyramidal neurons, especially in layers CA1 and CA3 of the hippocampus, and is associated with limited extra-hippocampal lesions^7^. In 2010, Norwood *et al*.^8^ developed an electrical stimulation paradigm in awake rats leading to the induction of hippocampal lesions with a morphology similar to that associated with human HS. More interestingly, these animals develop spontaneous seizures after a latent period of approximately 21 days without the presence of acutely induced generalized tonic clonic SE. To date, this is the only animal model that displays neuronal damage that more closely resembles HS present in patients with MTLE. Even though this animal model is well characterized, the molecular mechanisms that are ultimately responsible for epileptogenesis remain largely undetermined^8^,^9^.

Transcriptome profiling allows the broad mapping of molecular constituents present in cells and tissues, resulting in the possible generation of wide-ranging hypotheses about the mechanisms underlying physiological and pathological conditions^10^. With the advent of massive parallel sequencing of short DNA sequences, it is now possible to estimate the abundance of mRNA molecules in a sample, as well as determine its precise nucleotide sequence^10^. This strategy has advantages such as: that there is no probe design; and a larger linear range of quantity estimation^10^,^11^,^12^. Furthermore, after acquiring large mRNA data sets it is feasible to use this information to identify signaling pathways and biological processes most affected by a pathological condition by using the Gene Ontology system of classification of enriched genes.

The hippocampus is a highly heterogeneous structure, presenting different patterns of gene expression in different cell populations^13^,^14^. Furthermore, it is also possible to observe differences in gene expression even within the same cell population throughout the dorsal-ventral axis of the hippocampus^14^. Considering these characteristics, the precise isolation of cell populations to preserve spatial information significantly improves the specificity of the information obtained using high-throughput techniques to identify regional differences in molecular mechanisms.

Therefore, the main objective of the present study was to analyze, in one time point during disease progression, the transcriptome profile of the dorsal (dDG) and the ventral dentate gyrus (dDG) of animals with hippocampal lesions induced by Perforant pathway (PP) stimulation, and to explore the molecular mechanisms altered in this animal model.

## Results

**Figure 1:**
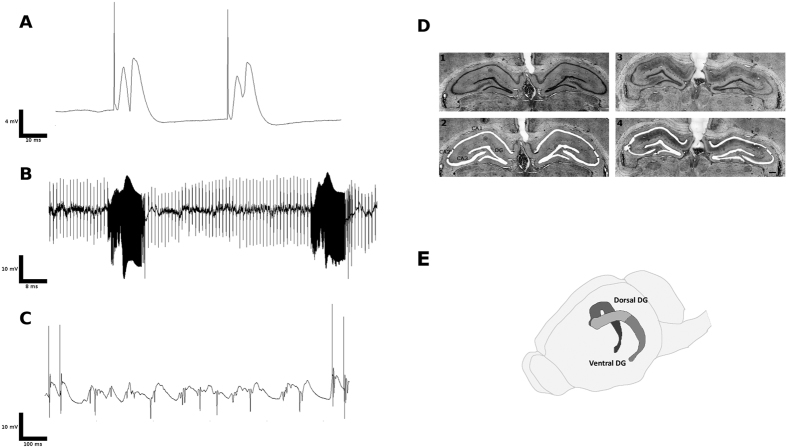
(**A**) Two evoked local field potentials in the DG after perforant pathway stimulation (40-ms interpulse interval) during electrode placement surgery. (**B**) Electrical recording of the DG during the perforant pathway stimulation protocol. (**C**) Detail of discharges observed after a 20-Hz train. (**D**) Nissl-stained sections used in the laser microdissection procedures. (**D**) 1 Section from control rat from the Sham group; (**D**) 2 Same section from control rat after microdissection; (**D**) 3 Section from an 8-h stimulated rat at 15 days after stimulation. Note extensive lesion in CA1 and CA3. (**D**) 4 Same section from stimulated rat after microdissection. Even though other hippocampus subfields were microdissected, only the DG was used in the present study. Scale bar = 500 μm. (**E**) Schematic drawing indicating the anatomical division adopted.

The stimulation of the PP during electrode placement surgery evoked depolarization of DG granular cells, and electrode positioning was adjusted for maximal amplitude of recorded depolarization. Furthermore, stimulation induced the occurrence of populational spikes (Fig. 1A). After a one-week recovery period, rats were stimulated for 30 minutes on two consecutive days and for 8 hours in the third day as previously described^8^. The stimulation induced epileptiform activity throughout the stimulation period (Fig. 1B,C). Control rats had electrodes implanted, but were not stimulated after the recovery period. Rats were video monitored after stimulation and did not present spontaneous seizures. Fifteen days after the last stimulation session, rats were euthanized, and brains were collected and processed for laser microdissection. In the present study, we selected the time point, of 15 days after induction of lesion by electrical stimulation because this consists in the ‘silent phase’ of the animal model used. We considered that a time point close to the 8 h electrical stimulation, as for example 48 h after induction, would present gene expression more associated with immediate response to electrical stimulation and apoptosis. A time point later, as for example 30 days would be in a phase were rats would already present spontaneous seizures, that could potentially induce changes in gene expression in the analyzed region due to seizures *per se*, and not necessarily due to the epileptogenesis process. Therefore, the time point chosen was sufficiently late in relation to the initial stimulation, but before the onset of spontaneous seizures; therefore reflecting molecular processes that most likely are involved in epilptogenesis.

Extensive pyramidal neuronal loss was observed in CA1, CA2 and CA3 in tissue sections from rats that were stimulated when compared to Sham controls (Fig. 1D). The dorsal and ventral regions of the DG granular cell layer were laser microdissected. In Fig. 1E we show a schematic drawing indicating the anatomical division adopted (Fig. 1E).

Total RNA was extracted from each sample, converted into cDNA libraries using Illumina TruSeq Stranded mRNA LT and sequenced in a HISeq^®^ 2500 platform. The sequencing run produced a total of 1,355,974,797 100-bp reads, with 94% of bases over Q30. An average of 84.7 million sequences were produced for each sample. Sequences were aligned with TopHat2 to *Rattus norvegicus* Ensembl Rnor 5.0 assemble. The average sequence alignment rate was 91.2%. HTSeqCount and DESeq2 packages were used for the estimation of gene expression and statistical analysis. After data processing, a list of differentially expressed genes with p < 0.05 (after correction for multiple tests) was generated. The EdgeR package was used to calculate the Biological coefficient of variance of the present data (BV = 0161), and the RNASeqPower^15^ package was used to calculate the statistical power of the present experiment, considering the number of biological replicates used. A power greater than 0.9 was found for genes that presented fold-change greater than 1,87 considering a depth of sequence of 20 and the Biological coefficient of variance calculated using the EdgeR package.

**Figure 2:**
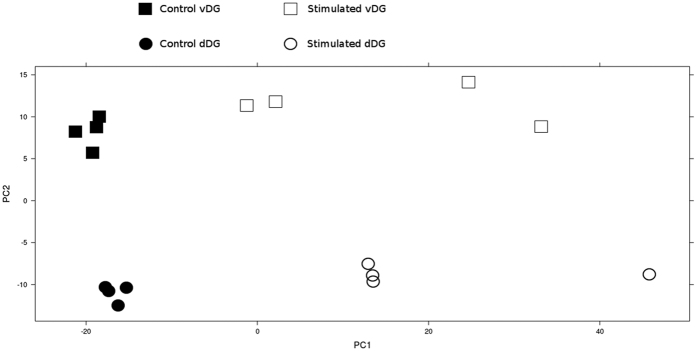
PCA graphic for gene expression data. It is possible to observe segregation between control and stimulated groups and between the regions of the dentate gyrus analyzed.

We found a total of 2,621 differentially expressed genes (p < 0.05) when comparing control dDG with stimulated dDG: 1,425 genes were up-regulated and 1,196 were down-regulated in stimulated samples. For the vDG we found 2,053 genes differentially expressed when comparing control vDG with stimulated vDG: 1,157 genes were up-regulated and 896 were down-regulated in the stimulated condition (*for a complete list refer to* supplementary Tables 1 and 2). The dimensionality reduction PCA analysis indicates a clear clustering of samples regarding the condition, control vs. stimulated, and in different regions of the DG, dDG vs. vDG (Fig. 2).

**Figure 3:**
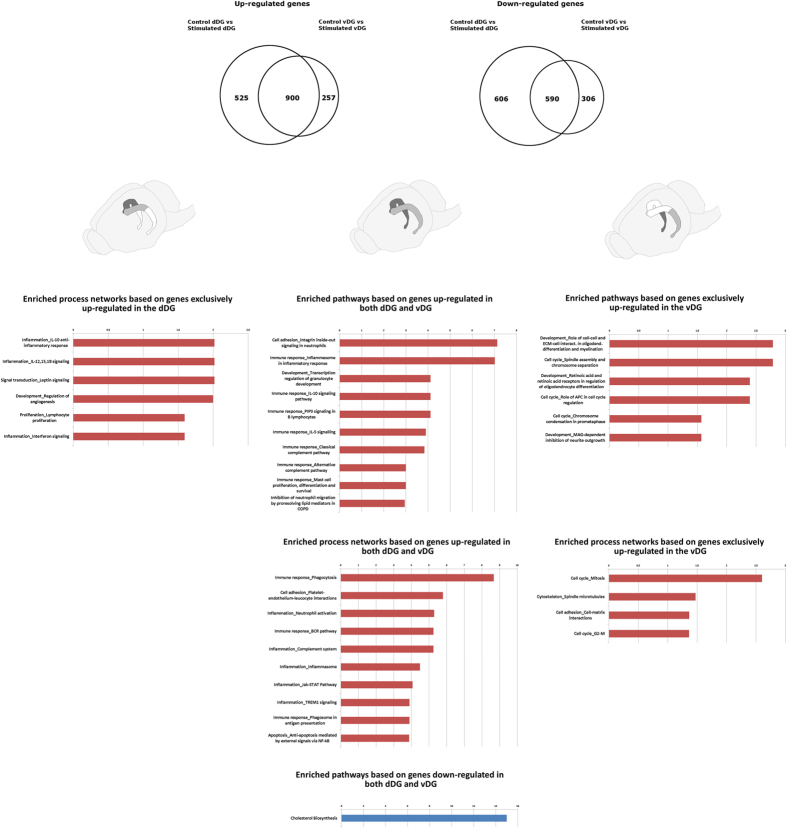
Venn diagram representing the number of differentially expressed genes. The left circle represents genes differentially expressed in the dorsal dentate gyrus (DG). The right circle represents genes differentially expressed in the ventral dentate gyrus, and the intersection contains differentially expressed genes common to both structures. Tables indicate gene onthologies enriched in each subset of genes represented in the Venn diagram. The X axis is the –log (adjusted p value).

Comparing the effect of stimulation on the dorsal and ventral regions of the DG, there were 1,490 differentially regulated genes in both regions: 590 up-regulated and 900 down-regulated. However, 1,131 genes were uniquely differentially regulated in the dDG: 607 up-regulated and 524 down-regulated. In addition, 563 genes were exclusively differentially regulated in the vDG: 306 up-regulated and 257 down-regulated as shown in Venn diagrams (Fig. 3). Furthermore, when directly comparing the dorsal to ventral portions of the DG from control rats a total of 2,449 genes were differentially expressed between regions, 1,142 up-regulated and 1,307 down-regulated. Comparing the dorsal to ventral portion of the DG from stimulated rats 2,133 genes were differentially expressed, 909 up-regulated and 1,224 down-regulated. The distribution of up and down-regulated genes in the dorsal and ventral regions was found to be homogenous as determined by a chi-square test (p = 0,1845).

We calculated the enrichment of pathways/biological processes for the set of differentially expressed genes using Metacore^®^ (Thomas-Reuter) (*for a complete list refer to* supplementary Tables 3 and 4). For the up-regulated genes in the dDG, 67 pathways and 53 biological processes were significantly enriched. Most significant pathways were those involved in immune response such as *Inflammosome in inflammatory response*, *IL-10 signaling*, *IL-5 signaling*, *classical complement pathway*, and processes such as *phagocytosis*, *inflammosome*, *interferon signaling* and *IL-10 inflammatory response*. For the down-regulated genes in the dDG, *cholesterol biosynthesis* was the only highly enriched pathway. This pathway had 16 of its 19 components down-regulated in the dDG of stimulated animals. For the up-regulated genes in the vDG, 45 pathways and 43 biological processes were significantly enriched. In the vDG, there was also a high frequency of immune response-related pathways such as *IL-10* and *-5 signaling*, *classical complement activation* and processes such as *phagocytosis* and *interferon signaling*. For the down-regulated genes in the vDG, *cholesterol biosynthesis* and *regulation of lipid metabolism* were the enriched pathways. However, two biological processes showed significant enrichments in the vDG: *calcium transport* and *axonal guidance*.

Enrichment pathways/biological process for genes, which were exclusively differentially regulated in stimulated animals were also identified (Fig. 3). For uniquely up-regulated genes in the dDG there were 14 enriched processes, all of which are involved in inflammatory response such as *IL-12*,*15*,*18 signaling*, *IL-10 anti-inflammatory response*, *interferon signaling*, *IL-6* and *leptin signaling*. Whereas, for the genes exclusively up-regulated in the vDG, there were six enriched pathways and four biological processes: cell cycle pathways such as *spindle assembly and chromosome separation*, *role of APC in cell cycle regulation*, *chromosome condensation in prometaphase*; cell cycle processes such as *mitosis G2-M* and *spindle microtubules*. Also enriched were the *cell adhesion – cell matrix interaction process* and *the role of cell-cell and extracellular matrix interaction in oligodendrocyte differentiation and myelination* and *MAG-dependent inhibition of neurite outgrowth*.

Enriched pathways and biological processes that did not reach statistical significance are shown in Table 1 (P > 0.05); however, they include sets of related genes that were significantly differentially regulated (corrected p values < 0.05 for individual genes).

A total of 18 genes that were identified as differentially regulated in the RNA sequencing experiments were selected for validation by real-time PCR relative quantification (Supplementary figure 1). These genes were selected either because they are components of the major molecular pathways found to be enriched in the transcritpome or because they are genes that were individually mentioned in the discussion. The results from the real-time PCR analysis confirmed the findings obtained by RNA sequencing for all 18 genes tested.

## Discussion

The present study explores for the first time the transcriptome of the hippocampus DG during the latent phase of an experimental epilepsy model presenting pathologic findings resembling classical human HS. Furthermore, due to the well-known spatial molecular diversity of the hippocampus^14^, we separately examined the dorsal and ventral sub-regions of the DG granular cell layer. Therefore, we were able to explore putative molecular mechanisms most likely involved in structural and functional changes that take place in the hippocampus in our model of mesial temporal lobe epilepsy, preserving spatial information about different neuronal circuits.

The most prevalent pathways and biological processes containing differentially expressed genes in both the dorsal and ventral regions of the DG were those related to the immune response. Moreover, the most enriched biological process associated with up-regulated genes in the electrically stimulated rats was phagocytosis. Even though neurons in the DG are considered a resistant cell population in the context of HS, some degree of cell loss within these granular neurons has been observed in humans^16^ and in animal models^17^. Interestingly, Norwood *et al*.^8^ did not describe any cell loss in the DG in this electrical stimulation model. However, the data presented by these authors did not exclude the possibility of some degree of cell loss because they did not quantify the cells or immunolabel infiltrating macrophage/microglial cells in the DG. Our data indicate a significant increase in expression of genes typically present in immune system cells specialized in phagocytosis, as indicated by the various enriched gene ontologies, such as *Inflammosome in inflammatory response* pathways or the *Phagocytosis* process. Furthermore, a typical marker of macrophage/activated microglial cells, *CD18* (also known as *CR3* or *ITGB2*)^18^ showed a 7.6- and 6.4-fold increase in expression in stimulated rats in the dDG and the vDG, respectively. The macrophage/microglia marker *Adgre1* (also known as *EMR1*)^19^ was also up-regulated in the dDG and vDG. Therefore, our results indicate a greater extent of damage in the dDG than previously reported^8^. This observation is supported by the immune system-related genes that were exclusively up-regulated in the dDG, such as those in the *JAK3/STAT5* pathway, and the morphological characteristics of the tissue observed by Nissl staining (Fig. 2). The observation of exclusive up-regulation in the dDG of *JAK3*, *STAT5*, *SOCS3* and genes related to *NFkβ* suggest a greater presence of immune system-related cells, such as lymphocytes infiltrating the dDG^20^,^21^. In an intrahippocampal kainic acid injection model in mice, an extensive degeneration of neurons is observed, which is associated with infiltrating microglia, circulating macrophages and lymphocytes in the degenerating hippocampus^22^. The infiltration of T lymphocytes in the dDG may be responsible for the observed up-regulation in the *JAK3/STAT5* pathway, since these immune cells are known to present a higher expression of these genes^20^,^21^. However, it is important to point out that although the up-regulation in immune system-related genes may be associated with the infiltration of immune system cells in the DG, it is possible that some of these genes may have been up-regulated in the granule cells. Additional experiments to better characterize the cellular composition of the DG during the latent phase of this electric stimulation epilepsy model, as well as the analysis of gene expression in isolated granule cells, may help to clarify the role of the immune system in the induction of HS and epileptogenesis.

The presence of various differentially regulated genes involved in axonal guidance such as class 3 Semaphorins, Ephrins and Integrins is noteworthy in the present study. Moreover, different members of these families of molecules are uniquely differentially regulated in the dorsal or the ventral portions of the DG. Semaphorins consist of a family of more than 20 secreted and membrane-bound proteins that may function as guidance cues during development and in the adult brain^23^,^24^,^25^. Beyond these established roles, Semaphorins were already shown to be differentially regulated in the hippocampus of experimental epilepsy models^26^. In the present dataset, we identified a high number of Semaphorins potentially involved in sprouting and abnormal circuitry formation. Furthermore, different Semaphorins (*Sema3a* and *Sema3b*, also known as Semaphorins 3A and 3B exclusively up-regulated in the dDG; *Sema3c* and *Sema5a* exclusively up-regulated in the vDG; *Sema3e* exclusively down-regulated in the dDG) were differentially regulated in different portions of the DG, indicating distinct axon guidance mechanisms functioning throughout the dorsal ventral hippocampal axis. Eph receptors and their respective ligands, the Ephrins, are also an important class of cell navigation and axon guidance molecules in the nervous system^27^. Therefore, these molecules may also be associated with molecular mechanisms underlying epileptogenesis as previously reported^28^.

An increase in neurogenesis of DG granule cells is a well-known phenomenon associated with epilepsy^29^. In the present dataset, we observed significantly enriched pathways related to cell proliferation only in the vDG, with the up-regulated genes involved in cell cycle control and mitosis. Furthermore, increased expression of the newborn neuronal marker *Dcx* (doublecortin) in both the dDG and vDG indicates the presence of migrating newly generated neuroblasts^30^. These observations may indicate an overall increase in neurogenesis, a phenomenon previously reported in the context of epileptogenesis^29^. In addition, our gene expression analyses indicated that this proliferation was more intense in the ventral region of the DG. Furthermore, we found an up-regulation of important genes related to myelination, such as the *Mag* (Myelin associated glycoprotein), *Mbp* (Myelin basic protein) and *Plp1*, present only in the vDG. This may indicate a greater extent of sprouting and rewiring taking place in the vDG, leading to an increase in the myelination process.

A common hypothesis for the induction of epileptogenesis is a change in the balance between excitation and inhibition in neural networks. It has been proposed that one possible mechanism for this change in balance would be a preferential loss of GABAergic neurons^31^,^32^. Another possibility would be a change in the expression pattern of GABA receptor subunits^33^,^34^. In the present dataset, we observed an up-regulation of some GABA A receptor subunits (such as *alpha 3* and *beta 2*) and a reduction of other subunits (such as the *delta* and *rho-3*) in both regions of the DG, as well as a down-regulation of the *Gabra5* (GABA A receptor alpha-5 subunit) in the dDG. Furthermore, changes in voltage-gated ion channels may also have an important role in the increase of excitability in the context of epileptogenesis. We observed an increase in the expression of *Scn4a* (Nav1.4), in both the dDG and vDG, a specific increase in *Scn9a* (Nav1.7) in the dDG and an increase in *Scn7a* (Nav2.1) in the vDG. The sodium channel isoform *Scn4a* is typically found in skeletal muscle^35^, whereas *Scn9a* is associated with dorsal root ganglia^36^. Thus, these channels are not usually associated with the hippocampus. The expression of these channel genes was low in the DG compared to other typical hippocampal sodium channels. For example, *Scn8a* (Nav1.6) had an average of 9,285 counts; whereas, *Scn4a* had an average of 285 counts and *Scn9a* an average of 154 counts. Nevertheless, these channels were significantly up-regulated in stimulated animals, suggesting an aberrant expression of sodium channels. Furthermore, their differential regulation may indicate some participation in the epileptogenic process. Voltage-gated potassium channels are a diverse family of genes that also have a key role in the regulation of neuronal excitability^37^. We found several voltage-gated potassium channel associated-genes differentially regulated in the stimulated animals. For example, expression of the gene *Kcnc2* (Kv3.2) was reduced in the dDG. It is worth noting that changes in the expression of such channels may have significant effects on neuronal firing, thus, rendering neurons more susceptible to seizures^38^.

Neuropeptides are an important class of molecules involved in the regulation of neural tissue excitability^39^. We observed changes in expression of neuropeptides and receptors, which can be of importance in the context of epilepsy. NPY is a neuropeptide with a significant role in the regulation of food-intake^40^, and it is associated with anticonvulsant activity^41^. Furthermore, intra-hippocampal administration of NPY is able to suppress seizures induced by perforant pathway stimulation^42^. Changes in the expression of NPY have previously been described in other experimental epilepsy models^43^, and the observed reduction in both the expression of NPY and two of its receptors (Y1 and Y5) in the dDG may play a role in a possible increase in excitability in this region. Interestingly, there was a concomitant reduction in the expression of other neuropeptides that may have an anticonvulsant role, such as *Pdyn* (also known as prodynorphin or proenkephalin B) a member of the opioid family^44^, and *Sst* (somatostatin) exclusively in the vDG^45^. It is worth noting that *Pdyn* is also associated with seizure suppression activity in the perforant pathway stimulation model^42^.

Nervous tissue contains high levels of cholesterol, and because cholesterol carrier proteins do not cross the intact blood brain barrier it is synthesized locally in the brain^46^. It is known that after a lesion, the level of cholesterol synthesis is reduced in nervous tissue, and cholesterol is recycled from degenerating terminals^47^. In the hippocampus, for example, damage to the enthorinal cortex leads to extensive degeneration of synaptic input to the DG, resulting in reduction of hippocampal cholesterol synthesis and an increase in the uptake of cholesterol from degenerating terminals via *ApoE*^47^. In epilepsy models, a change in cholesterol metabolism due to epilepsy induction was previously observed^48^. Our results show that the most significantly down-regulated process in both regions of the DG was cholesterol synthesis. Almost all enzymes participating in this process showed reduced expression. These observations indicate the presence of degenerating axons and dendrites in the DG, hence possibly triggering the recycling of cholesterol from such degenerating neuronal projections.

## Conclusions

The transcriptome data produced and analyzed in the present study reveal the functional profiles and possible components of the molecular mechanisms that most likely are involved in epileptogenesis in an animal model that displays HS. The gene ontologies enriched in the present dataset implicate several biological processes such as immune-response, the interplay between neural and immune systems, cell death, neural circuitry re-wiring, changes in cell excitability, neurotransmission and metabolic changes. All of these processes are likely to play a role, probably simultaneously, in the induction of epileptogenesis. Of particular interest is the possibility of interplay between the immune system and the granule neurons. This may involve signaling pathways, trophic molecules, cell migration and cell projection guidance molecules, such as those that attract and guide the migration of immune-system cells. These mechanisms may change the migration and projection pattern of neural cells, contributing to the generation of ictogenic tissue.

Many individual processes mentioned above have been previously associated to some degree with epileptogenesis. However, the most important contribution of the present study is that by generating a large and comprehensive dataset, one can more precisely identify specific molecular components of the many biological systems likely contributing to the structural and functional changes that take place in the damaged hipppocampus. Furthermore, the present data also indicate that even though similar mechanisms may be found in different regions of the DG, the components involved seem to be region specific. Finally, our results identified a large number of novel potential targets that may ultimately help inhibit the epileptogenic process.

## Methods

### Animals

In the present study, three-month-old male Wistar rats (n = 8) were used. Rats were housed under a 12 h/12 h light cycle on a ventilated rack with *ad libitum* access to standard rodent chow and water. All procedures were performed in accordance with the ethical standards for animal experimentation at the University of Campinas-UNICAMP. The experimental protocol was approved by the UNICAMP animal research ethics committee, which evaluates experimental protocols according to current accepted ethical practices and legislation regarding animal research in Brazil (Brazilian federal law 11.794 (10/08/2008).

### Electrode placement surgery and stimulation

Rats were anaesthetized with an isoflurane/oxygen mixture (2%/98%) at 2 L/min using an acrylic induction box. We maintained anesthesia throughout the entire surgical procedure with the aforementioned mixture and administration rate by using a mask adapted to a stereotaxic apparatus. After a deep anesthesia state was verified by the lack of pedal and corneal reflex, we positioned the animal on a stereotaxic apparatus (Leica, Angle Two). The skull was exposed by an incision on the scalp, the Bregma was localized and bone perforations were performed in the following coordinates: +4.5 mm lateral, −7.6 mm posterior; −4.5 mm lateral, −7.6 mm posterior; +2 mm lateral, −3 mm posterior and −2 mm lateral, −3 mm posterior. We performed three perforations in the spaces between the aforementioned coordinates for the fixation of stainless steel screws. One additional perforation was performed 2 mm posterior to the lambdoid suture, in the midline for the placement of a reference electrode (stainless steel, polymide covered, 0.25 mm, Plastics One), the reference electrode was lowered just over the surface of the dura mater.

Bipolar stimulation electrodes (stainless steel, polymide covered, 0.125 mm, Plastics One) with a 1-mm separation were positioned into the perforant pathway (+−4.5 mm lateral, −7.6 mm posterior, −3 mm ventral). Monopolar recording electrodes (stainless steel, polymide covered, 0.25 mm, Plastics One) were positioned into the DG (+−2 mm lateral, −3 mm posterior, −3.5 mm ventral). We optimized the final positioning of the recording and stimulation electrodes based on polarity, occurrence of population spike and maximum amplitude of evoked potentials in the DG (Fig. 1A)^49^. We performed electrical stimulations with a Grass Astro-Med S88 stimulus generator (paired pulses, 0.1-ms pulse duration, interpulse interval of 40 ms, and pulse amplitude of 20 V). Recordings were performed with a miniature pre-amplifier (ThomasRecording^®^, 2 channel, 10× gain, dc coupled, 0.06 Hz high-pass filter, 10 kHz low-pass filter) connected to a main amplifier (ThomasRocording^®^, 8 channel, 25× gain, 0.06 Hz high-pass filter, 10 kHz low-pass filter), and the signal was digitalized at 10 kHz (NIDaq). After electrode and screw positioning, dental acrylic cement was applied in order to hold and stabilize all elements.

Seven days after surgery of electrode placement, freely moving awake rats were stimulated as described previously^8^ briefly, two days with 30-min stimulation sessions, followed by an 8-h session on the third day. The following stimulation protocol was used during stimulation: continuous 2-Hz paired pulse, 40-ms interpulse interval stimulation, and once per minute a 10-s, 20-Hz single pulse train was delivered using a Grass^®^ Astro-Med S88 stimulator. We recorded DG field potentials throughout the stimulation with the same configuration described for the electrode placement surgery (Fig. 1B,C). Control rats (n = 4) also had surgery for electrode implantation, they were handled and recorded using the same protocols as stimulated rats, but they did not receive any electric stimulation during or after the surgery of electrode placement.

Fifteen days after the last stimulation session, rats were deeply anaesthetized with an isoflurane/oxygen mixture (2%/98%). They were quickly decapitated and the brain was immediately removed and frozen at −60 °C using dry ice and n-hexane.

### Laser microdissection

We processed previously frozen brains in a cryostat in order to obtain 60-μm serial sections covering the entire hippocampus. Subsequently, we mounted tissue sections in PEN membrane covered slides (Life Technologies^®^). These, were immediately stained with Cresyl Violet, dehydrated with an ethanol series and stored at −80 °C. The DG was divided into dorsal and ventral regions according to previously described morphological criteria^14^. The dDG and the vDG were delimited with a Palm (Zeiss^®^) system, and tissue was mechanically collected in separate tubes using a surgical microscope and micro-forceps (Fig. 2)

### cDNA library preparation and next-generation sequencing

We extracted RNA from microdissected tissue samples with Trizol^®^, using the manufacturer instructions. cDNA libraries were produced from 200 ng of extracted RNA using the TruSeq Stranded mRNA LT (Illumina^®^) according to the manufacturer instructions. Each sample had different identifier barcodes, which allowed many samples to be sequenced in the same lane. We sequenced cDNA libraries in a HiSeq^®^ 2500 (Illumina^®^) in High Output mode, producing 100-bp, single-read sequences. The sequencing run produced a total of 1,355,974,797 100-bp reads, with 94% of bases over Q30. An average of 84.7 million of sequences was produced for each sample. We aligned sequences with TopHat2 (http:// http://ccb.jhu.edu/software/tophat/index.shtml) to *Rattus norvegicus* Ensembl Rnor 5.0 assemble. The average sequence alignment rate was 91.2%. The HTSeqCount and DESeq2 packages (http://www-huber.embl.de/users/anders /HTSeq/doc/overview.html, as well as http://www.bioconductor.org/ packages/release/bioc/html/DESeq2.html) were used for the estimation of gene expression and statistical analysis. HTSeqCount performs the counting of aligned sequences to genome elements, such as exons and genes. DESeq2 employs a negative-binomial distribution for data normalization, corrects for outliers and the contribution of low expression genes for the analysis of variance, and it uses differential expression statistical Wald tests and corrects for multiple tests employing the Benjamini and Hochberg procedures. After data processing, a list of differentially expressed genes with a statistical significance set at a p < 0.05 (after correction for multiple tests) was generated. We used the differentially expressed genes list for gene ontology analysis with the Metacore^®^ software (Thomas-Reuter).

### Gene expression analysis by real time RT-PCR

Total RNA that was previously extracted from microdissected tissue samples (200 ng per sample) was used for cDNA synthesis using Vilo cDNA synthesis kit (Thermo^®^) following the manufacturer instructions. Relative gene expression was analysed using the 2^−ΔctΔct^ method, for this analysis, primers pairs for the genes: *c3, cd18, socs3, mbp, npy, snc4a, sema3a, sema3c, dcx, stat5, adger1, apoe, cdk1, cyp51a1, fdft1, pdyn, sst* and *y1*, were designed using NCBI primer design tool (http://www.ncbi.nlm.nih.gov/tools/primer-blast/). As endogenous controls the geometric mean of the *ct* of the genes *ssr2, ankyf* and *smntl2* were used. For the PCRs, the Sybr Power Plus Master Mix kit (Thermo^®^) was used following the manufacturer instructions in a Applied 7500 real-time PCR equipment (Applied Biosystems^®^). Following PCR amplifications, a melting curve was analysed for each gene, and a single TM peak was observed.

## Additional Information

**How to cite this article**: Vieira, A. S. *et al*. RNA sequencing reveals region-specific molecular mechanisms associated with epileptogenesis in a model of classical hippocampal sclerosis. *Sci. Rep*. **6**, 22416; doi: 10.1038/srep22416 (2016).

## Supplementary Material

Supplementary Table Legends

Supplementary Figures

Supplementary Table S1

Supplementary Table S2

Supplementary Table S3

Supplementary Table S4

Supplementary Table S5

Supplementary Table S6

## Figures and Tables

**Table 1:** Relevant genes that were differentially regulated in the dentate gyrus (DG) of stimulated animals in the context of epileptogenesis.

Axon guidance	
Common up-regulated genes in the dDG and vDG	*dihydropyrimidinase-like 3*, *dihydropyrimidinase-like 5*, *Ephrin-A5*, *EVL*, *integrin beta 2*, *LIM domain kinase 1Semaphorin 3D* and the *vasodilator-stimulated phosphoprotein*.
Exclusively up-regulated in the dDG	*dihydropyrimidinase-like 1, Ephrin-A receptor 1, Ephrin-A1, IQGAP1, myosin regulatory light chain interacting protein*, *plexin-B2*, *Semaphorin 3A*, *Semaphorin 3B*.
Exclusively up-regulated in the vDG	fasciculation and elongation protein zeta 2, *integrin beta 1*, *nerve growth factor receptor, Semaphorin 3C, Semaphorin 5A*
Common down-regulated genes in the dDG and vDG	*Ephrin-A receptor 8*, *Ephrin-B1*, *Ephrin-B2*, *Ephrin-B3*, *Guanine deaminase*, *Plexin A3*, *Plexin C1, slit homolog 2*, *Ephexin*, *Ryanodine receptor 1*
Exclusively down-regulated in the dDG	*NCK-associated protein 1, reticulon 4*, *Semaphorin 3E*
Exclusively up-regulated in the vDG	reelin signal transducer, amyloid beta (A4) precursor protein-binding, growth arrest-specific 7, roundabout axon guidance receptor homolog 3, *Semaphorin 7*.
Synaptic function	
Common up-regulated genes in the dDG and vDG	GABA-A receptor alpha-3 subunit, GABA-A receptor beta-2 subunit, Synaptotagmin VI, contactin 1, TANC1, Ephrin-A5, WNT3A, syntaxin binding protein 2, solute carrier family 6 (neurotransmitter transporter), member 5, growth factor receptor-bound protein 2, beta-2 adrenoreceptor, glutamate receptor ionotropic delta 2 (GluD2), cannabinoid receptor 2 (CNR2),
Exclusively up-regulated in the dDG	solute carrier family 17 member 6, EPH receptor B6, Semaphorin 4C, homer homolog 3, adrenoceptor alpha 2C
Exclusively up-regulated in the vDG	GABA-A receptor epsilon, GABA-A receptor alpha-1 subunit, cholinergic receptor, nicotinic, beta 4 (neuronal)
Common down-regulated genes in the dDG and vDG	BEGAIN, Ephrin-B1, B2 and B3, Homer 1, Syntrophin gamma 2, TRPC5, VAMP1, WNT7A, GABA-A receptor delta subunit, Cerebellin 1, GABA receptor rho-3, GluR7, cholinergic receptor nicotinic alpha 7 (CHRNA7), histamine receptor H3,
Exclusively down-regulated in the dDG	fibroblast growth factor receptor substrate 2, N-ethylmaleimide-sensitive factor attachment protein alpha, GABA-A receptor alpha-5 subunit, limbic system-associated membrane protein, Glycine receptor beta chain
Exclusively up-regulated in the vDG	Calmodulin, Dab, reelin signal transducer homolog 1, DLGAP3, MAGI-1, nAChR alpha-4, Neurogranin, Synaptotagmin X, SAP102, adrenoceptor beta 1, glutamate receptor, metabotropic 6.
Neuronal electrical activity	
Common up-regulated genes in the dDG and vDG	potassium voltage-gated channel subfamily E member 1-like protein (KCNE1L), potassium voltage-gated channel, subfamily H member 2 (KCNH2), P2 × 7, Na(V)1.4,
Exclusively up-regulated in the dDG	Nav1.7, potassium voltage-gated channel Shaw-related subfamily member 4 (KV3.4),
Exclusively up-regulated in the vDG	P2 × 1, Nav2.1, voltage-dependent calcium channel protein TPC2, potassium intermediate/small conductance calcium-activated channel subfamily N member 1,
Common down-regulated genes in the dDG and vDG	Navbeta4 (SCN4B), contactin associated protein 1
Exclusively down-regulated in the dDG	KCNMB2, KCNC2 (Kv3.2), connexin 36
Exclusively up-regulated in the vDG	KCNMB4, CACNA1H (Cac3.2), calcium channel, voltage-dependent L type alpha 1S subunit (CACNA1S)
Neuropeptides	
Common up-regulated genes in the dDG and vDG	growth factor receptor-bound protein 2, neuropeptide Y receptor Y2 (NPY2R), SHC1, EMR1, prolactin releasing hormone receptor,
Exclusively up-regulated in the dDG	corticotropin releasing hormone receptor 1, tachykinin 3, prokineticin receptor 2,
Exclusively up-regulated in the vDG	neuromedin U,
Common down-regulated genes in the dDG and vDG	Calcitonin, Neuromedin B, Orexin receptor 2, Proenkephalin-B, somatostatin receptor 4
Exclusively down-regulated in the dDG	gastrin-releasing peptide (proGRP), NPY, NPY1R, NPY5R, Somatostatin, adenylate cyclase activating polypeptide 1
Exclusively up-regulated in the vDG	Substance P
Neuronal proliferation and migration	
Common up-regulated genes in the dDG and vDG	SLIT-ROBO Rho GTPase activating protein 2 (SRGAP2), Doublecortin, DYLX2 , wingless-type MMTV integration site family, member 7B (WNT7B), neuronatin,
Exclusively up-regulated in the dDG	
Exclusively up-regulated in the vDG	cell adhesion molecule L1-like (CHL1), MET proto-oncogene receptor tyrosine kinase (HGF receptor),
Common down-regulated genes in the dDG and vDG	BTG family member 2 (BTG2), doublecortin domain containing 2 (DCDC2) FAT atypical cadherin 3 (FAT3), fibroblast growth factor 13, chemokine (C-X-C motif) ligand 12
Exclusively down-regulated in the dDG	scratch family zinc finger 1 (SCRT1), zinc finger, SWIM-type containing 6 (ZSWIM6)
Exclusively up-regulated in the vDG	ROBO3, Pro-CCK, serum response factor (SRF), amyloid beta precursor protein-binding family B member 1 (Fe65)

Genes are grouped by function and DG region.
