# Supplementary material for: RNA sequencing reveals region-specific molecular mechanisms associated with epileptogenesis in a model of classical hippocampal sclerosis
Source: Sci Rep. 2016 Mar 3;6:22416. doi: 10.1038/srep22416 (PMC4776103; doi:10.1038/srep22416)
Supplement: Supplementary Table Legends [file srep22416-s1.doc]

**RNA sequencing reveals region-specific molecular mechanisms associated with epileptogenesis in a model of classical hippocampal sclerosis**

Vieira AS1*, de Matos AH1*, do Canto AM1*, Rocha CS1*, Carvalho B1*, Pascoal VDB2*, Norwood B3, Bauer S3, Rosenow F3, Glioli R4, Cendes F5*, Lopes-Cendes I1*

**Supplementary Information**

**Supplementary table 1 –** List of genes differentially regulated in the dorsal dentate gyrus when comparing control to stimulated rats.

**Supplementary table 2 –** List of genes differentially regulated in the dorsal dentate gyrus when comparing control to stimulated rats.

**Supplementary table 3 –** List of pathways and biological process significantly enriched based on up or down-regulated genes in the dorsal dentate gyrus.

**Supplementary table 4 –** List of pathways and biological process significantly enriched based on up or down-regulated genes in the ventral dentate gyrus.

**Supplementary table 5 –** List of genes differentially regulated in control rats when comparing the dorsal to the ventral dentate gyrus.

**Supplementary table 6 –** List of genes differentially regulated in stimulated rats when comparing the dorsal to the ventral dentate gyrus.

**Supplementary data 1** – RNAseq and Real-time RT-PCR gene expression data for 18 selected genes.
