# Supplementary material for: RNA sequencing reveals region-specific molecular mechanisms associated with epileptogenesis in a model of classical hippocampal sclerosis
Source: Sci Rep. 2016 Mar 3;6:22416. doi: 10.1038/srep22416 (PMC4776103; doi:10.1038/srep22416)
Supplement: Supplementary Figures [file srep22416-s2.doc]

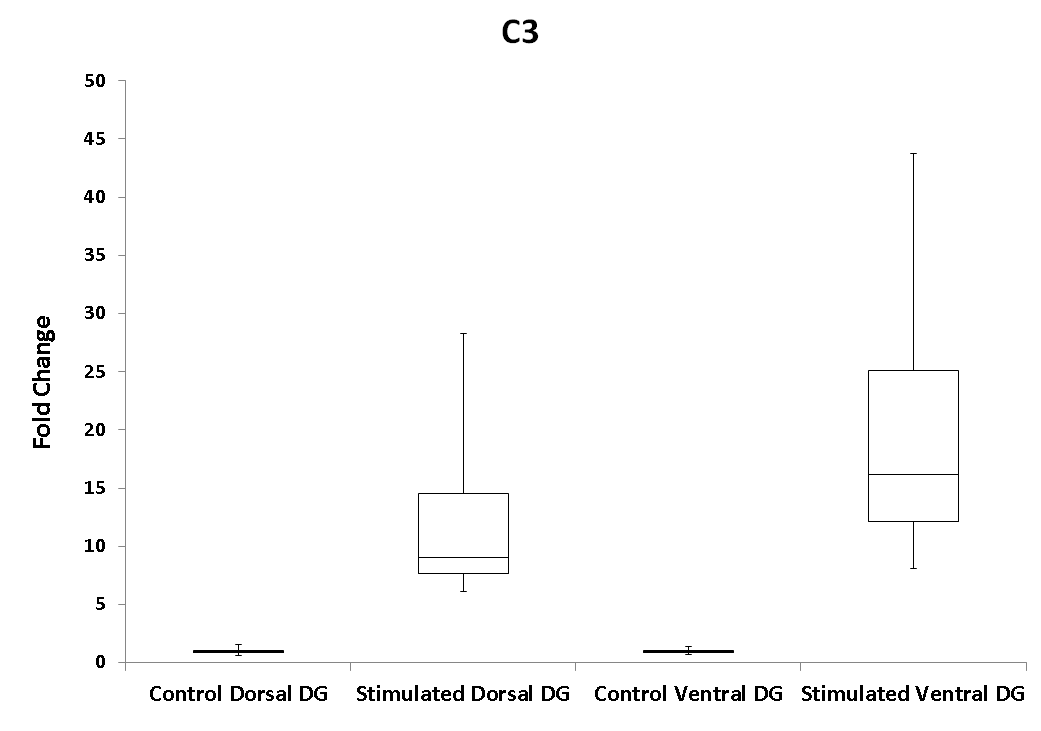


**A**

**B**


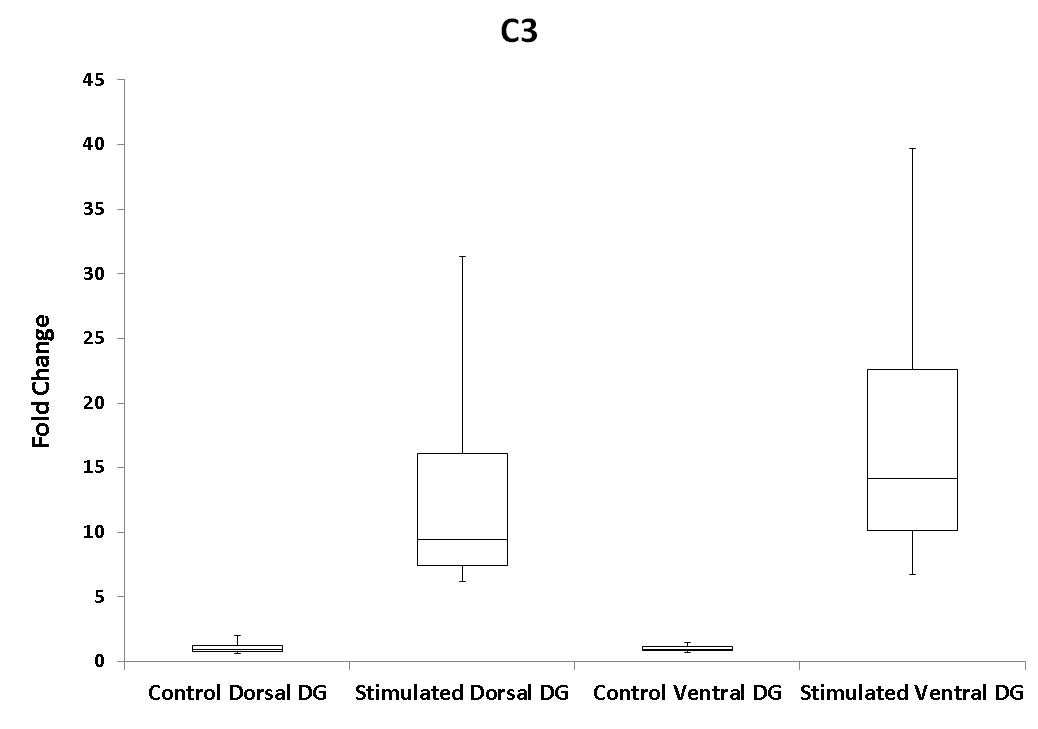


**Supplementary Figure 1.1** – Box plots of RNASeq and Real-Time PCR expression data for the *c3* gene. **A** – RNASeq normalized count data from DESEQ2. Statistical data from DESEQ2: Control vs Stimulated Dorsal DG p-value < 0.0001; Control vs Stimulated Ventral DG p-value < 0.0001. **B** – Real-Time PCR relative quantification data. T-Test: Control vs Stimulated Dorsal DG p-value = 0.068; Control vs Stimulated Ventral DG p-value = 0.052.


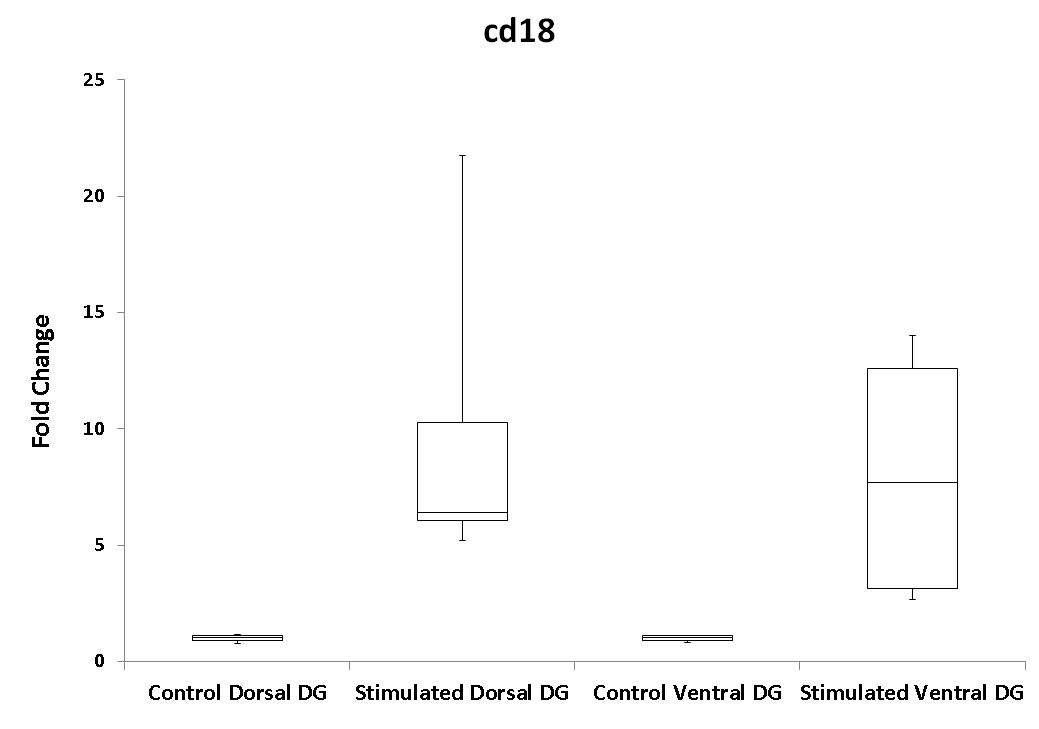


**A**


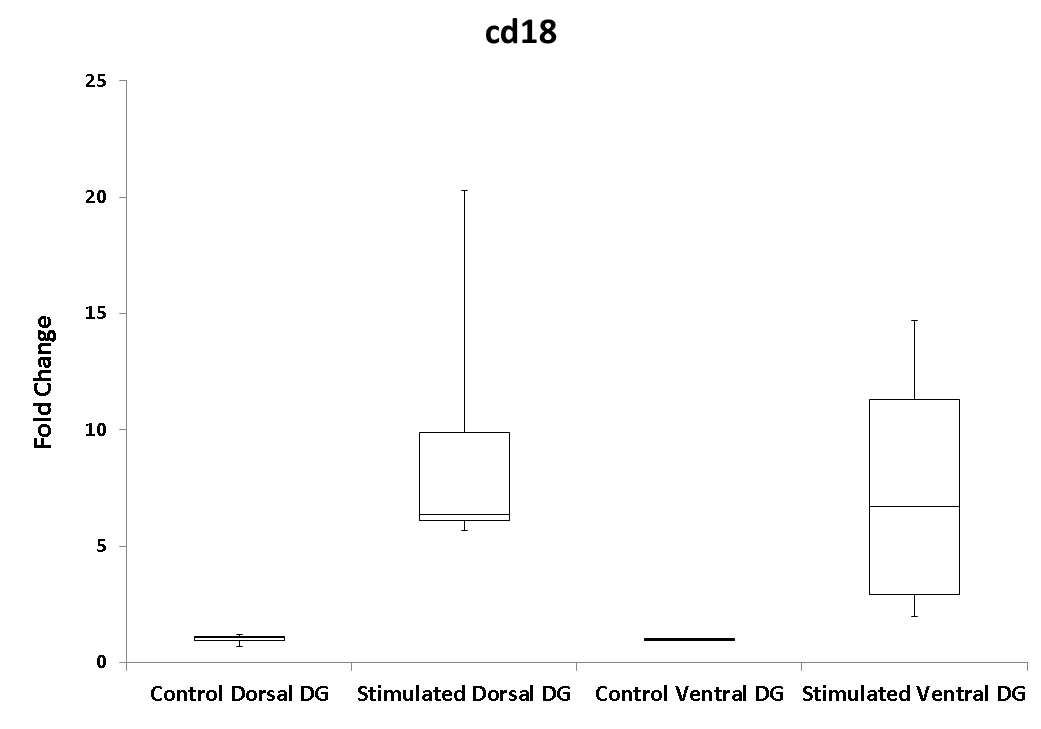


**B**

**Supplementary Figure 1.2** – Box plots of RNASeq and Real-Time PCR expression data for the *cd18* gene. **A** – RNASeq normalized count data from DESEQ2. Statistical data from DESEQ2: Control vs Stimulated Dorsal DG p-value < 0.0001; Control vs Stimulated Ventral DG p-value < 0.0001. **B** – Real-Time PCR relative quantification data. T-Test: Control vs Stimulated Dorsal DG p-value = 0.051; Control vs Stimulated Ventral DG p-value = 0.072.


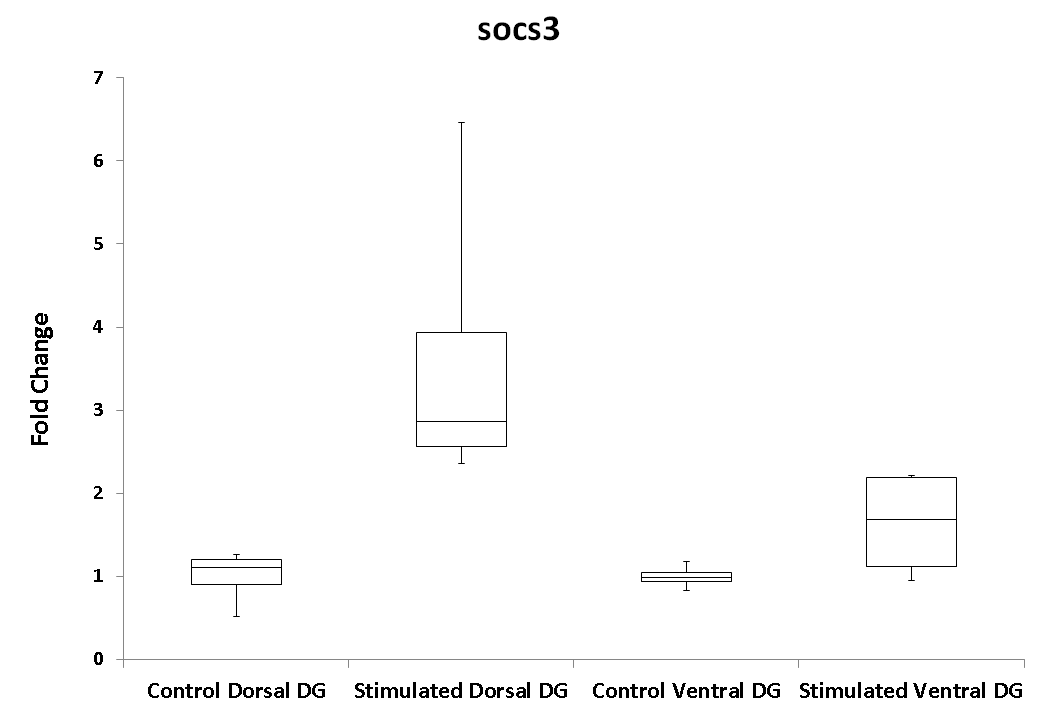


**A**


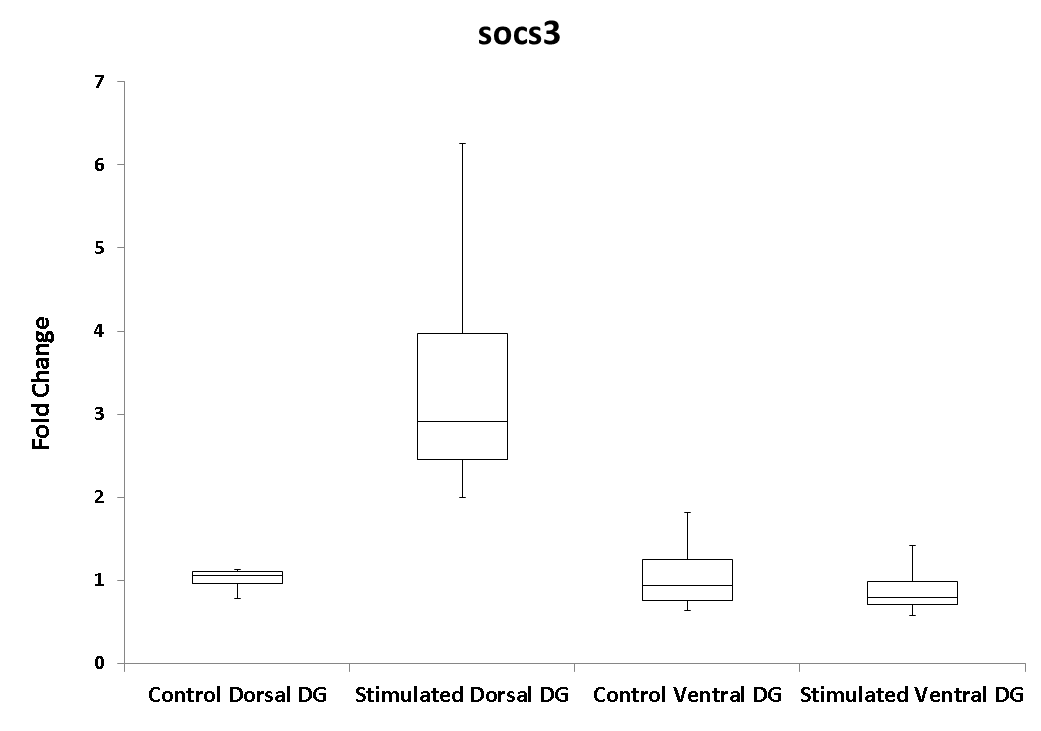


**B**

**Supplementary Figure 1.3** – Box plots of RNASeq and Real-Time PCR expression data for the *socs3* gene. **A** – RNASeq normalized count data from DESEQ2. Statistical data from DESEQ2: Control vs Stimulated Dorsal DG p-value = 0.0004; Control vs Stimulated Ventral DG p-value = 0.315. **B** – Real-Time PCR relative quantification data. T-Test: Control vs Stimulated Dorsal DG p-value = 0.038; Control vs Stimulated Ventral DG p-value = 0.583.


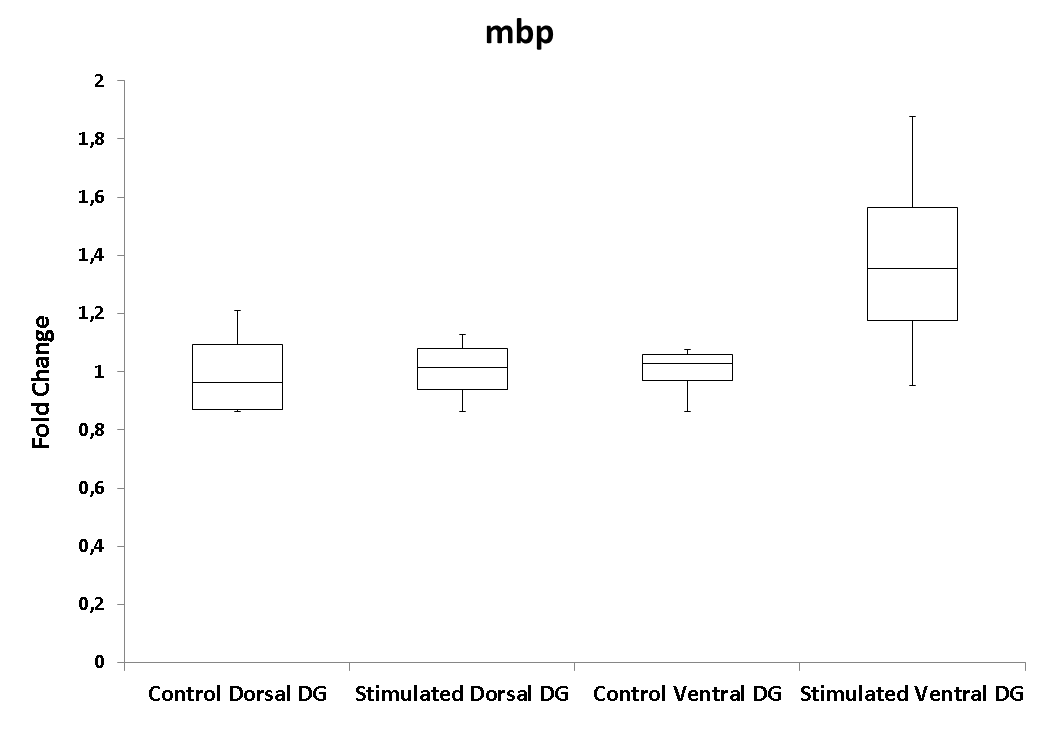


**A**


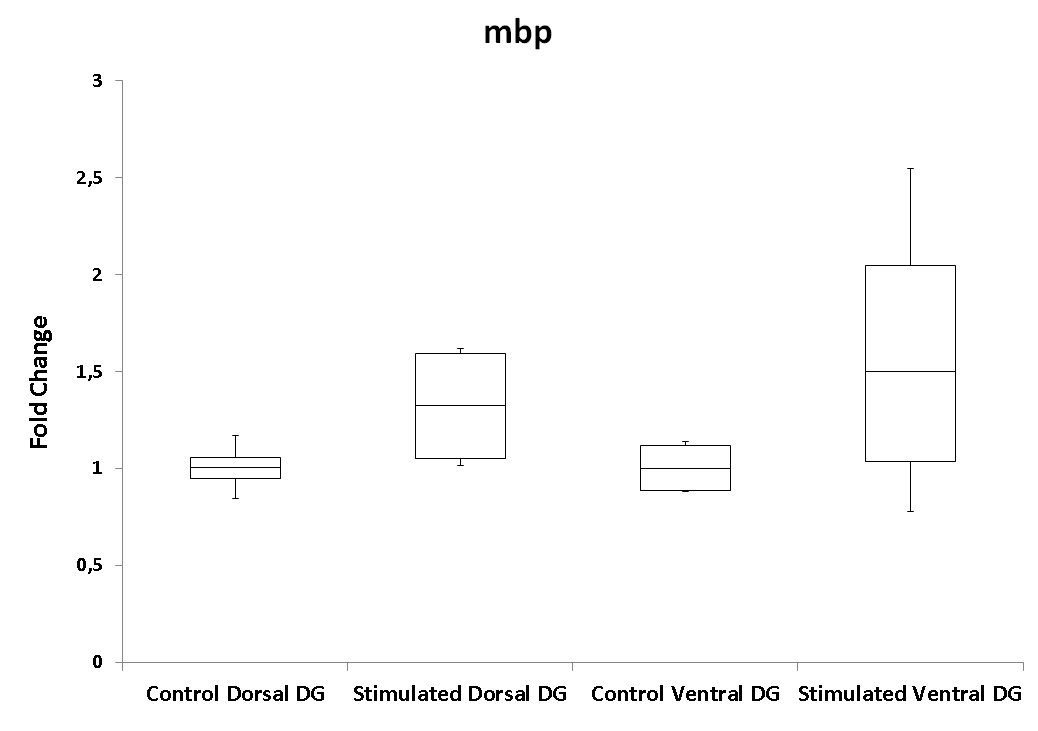


**B**

**Supplementary Figure 1.4** – Box plots of RNASeq and Real-Time PCR expression data for the *mbp* gene. **A** – RNASeq normalized count data from DESEQ2. Statistical data from DESEQ2: Control vs Stimulated Dorsal DG p-value = 0.986; Control vs Stimulated Ventral DG p-value = 0.041. **B** – Real-Time PCR relative quantification data. T-Test: Control vs Stimulated Dorsal DG p-value = 0.123; Control vs Stimulated Ventral DG p-value = 0.202.


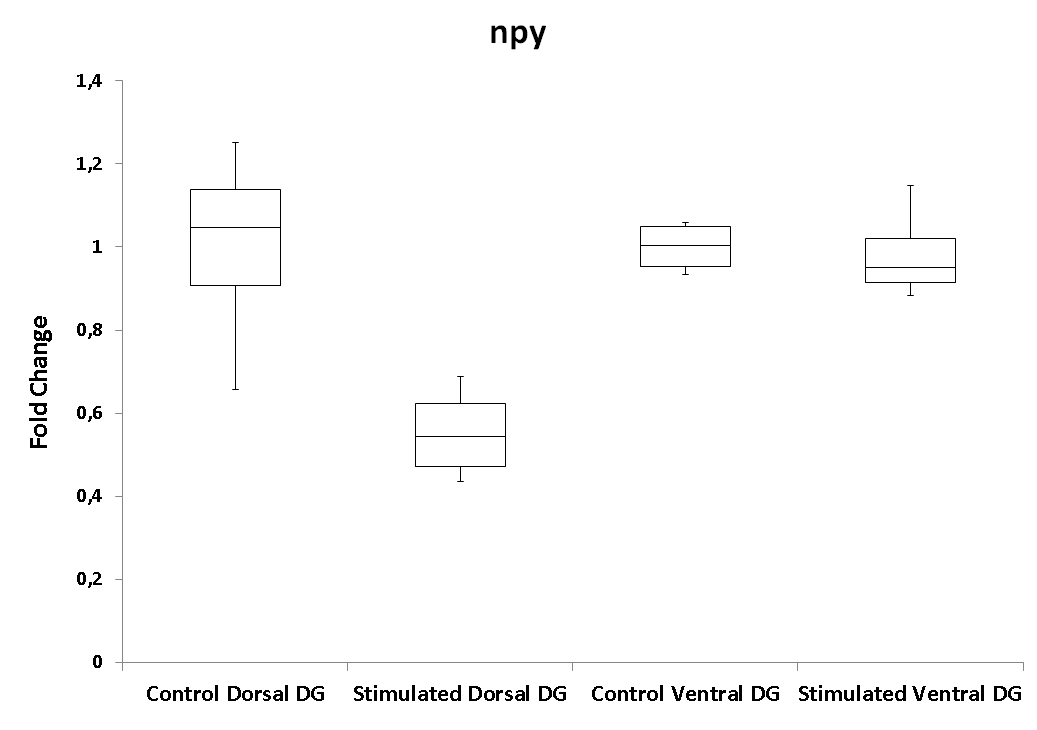


**A**

**B**


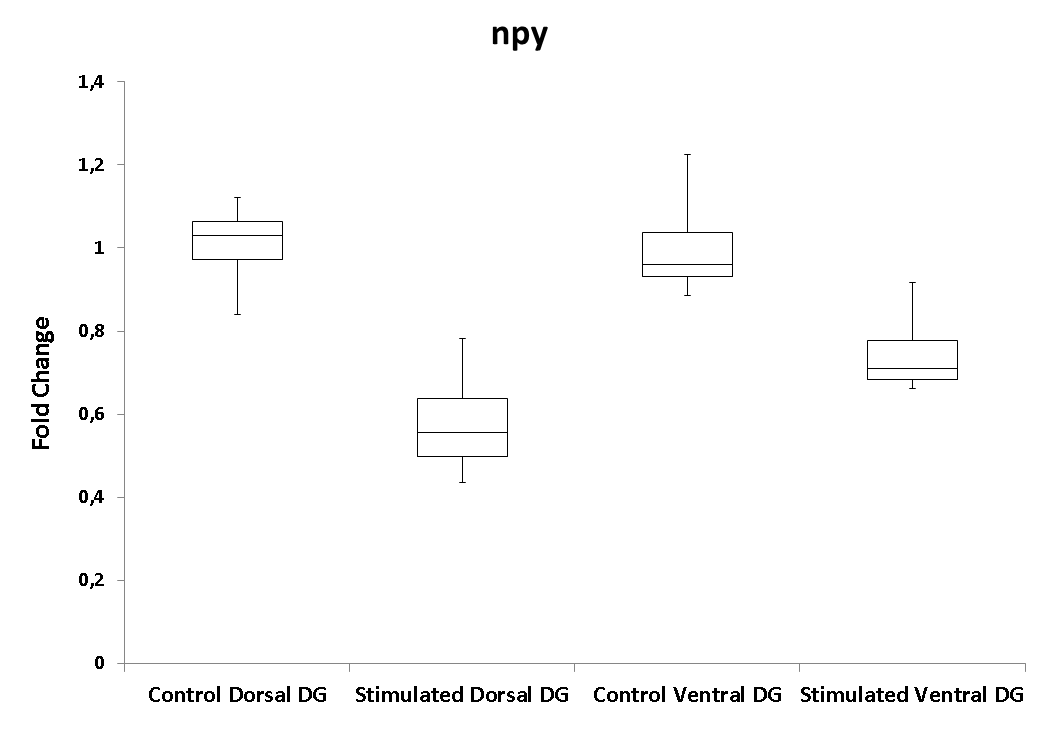


**Supplementary Figure 1.5** – Box plots of RNASeq and Real-Time PCR expression data for the *npy* gene. **A** – RNASeq normalized count data from DESEQ2. Statistical data from DESEQ2: Control vs Stimulated Dorsal DG p-value = 0.0003; Control vs Stimulated Ventral DG p-value = 0.971. **B** – Real-Time PCR relative quantification data. T-Test: Control vs Stimulated Dorsal DG p-value = 0.004; Control vs Stimulated Ventral DG p-value = 0.034.


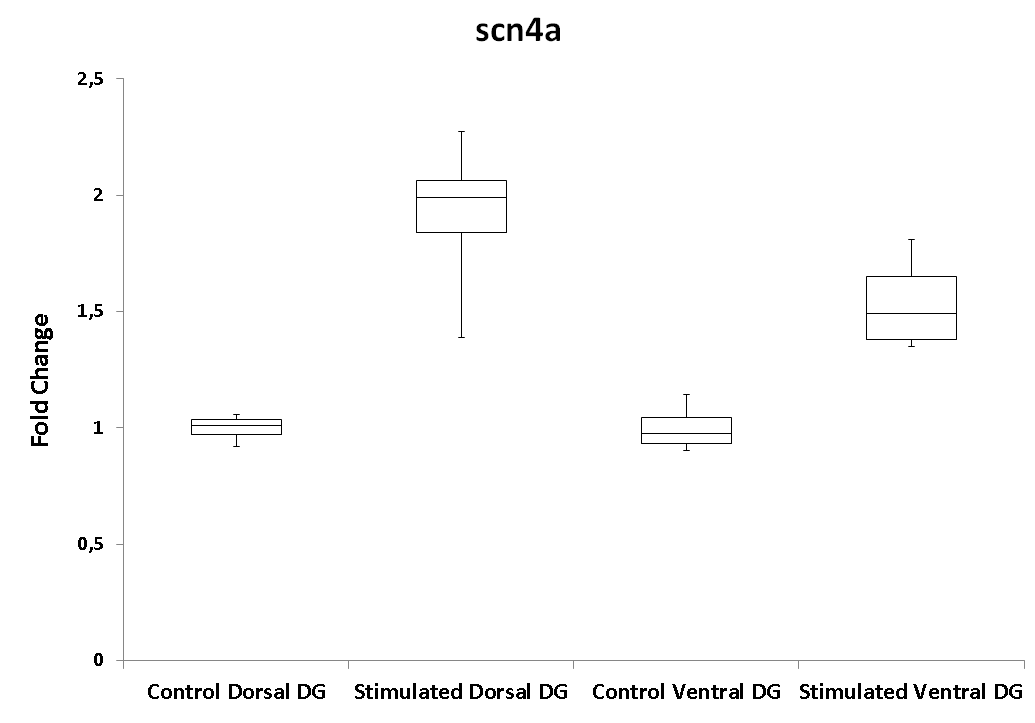


**A**

**B**


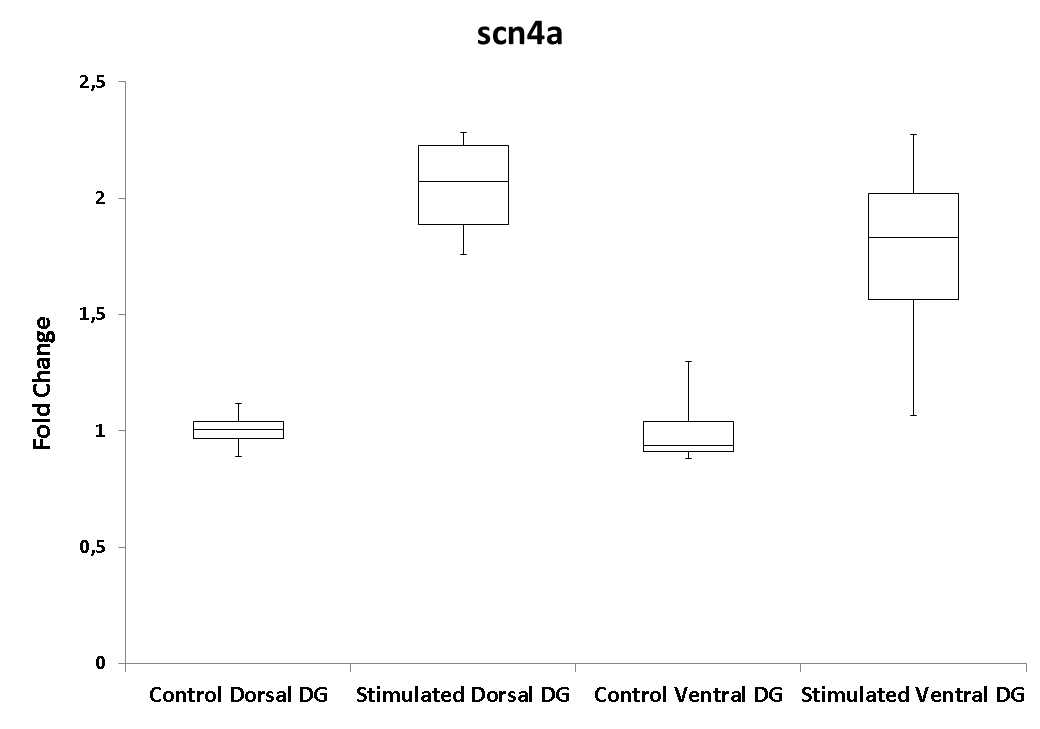


**Supplementary Figure 1.6** – Box plots of RNASeq and Real-Time PCR expression data for the *scn4a* gene. **A** – RNASeq normalized count data from DESEQ2. Statistical data from DESEQ2: Control vs Stimulated Dorsal DG p-value < 0.0001; Control vs Stimulated Ventral DG p-value = 0.0003. **B** – Real-Time PCR relative quantification data. T-Test: Control vs Stimulated Dorsal DG p-value = 0.0002; Control vs Stimulated Ventral DG p-value = 0.035.


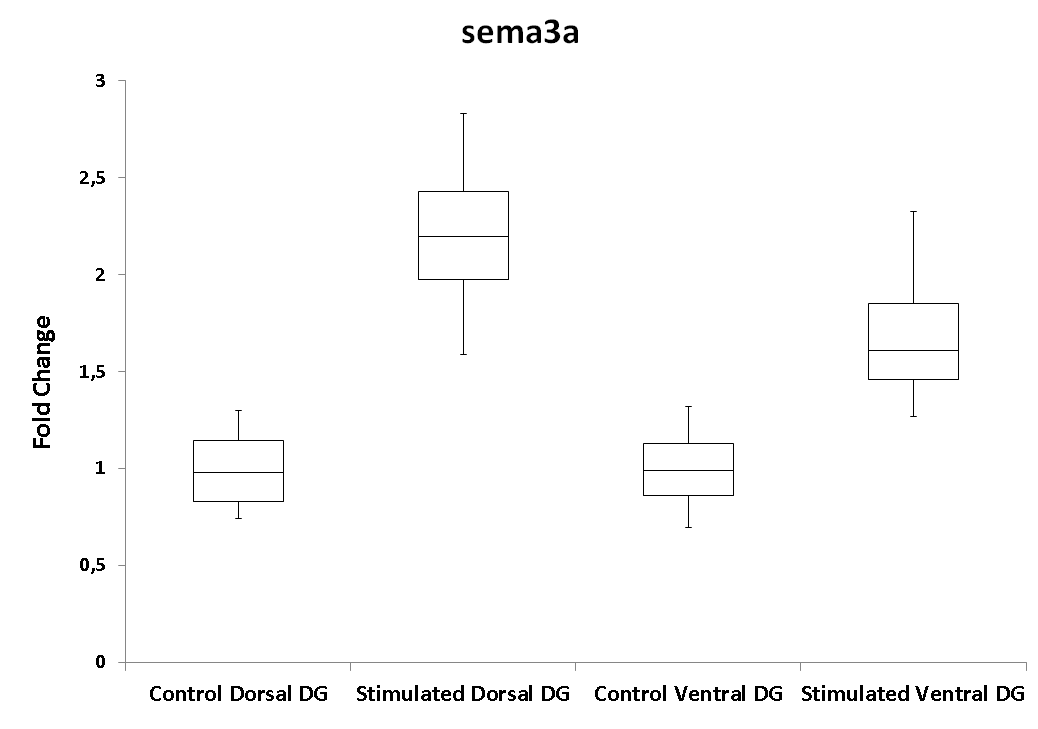


**A**

**B**


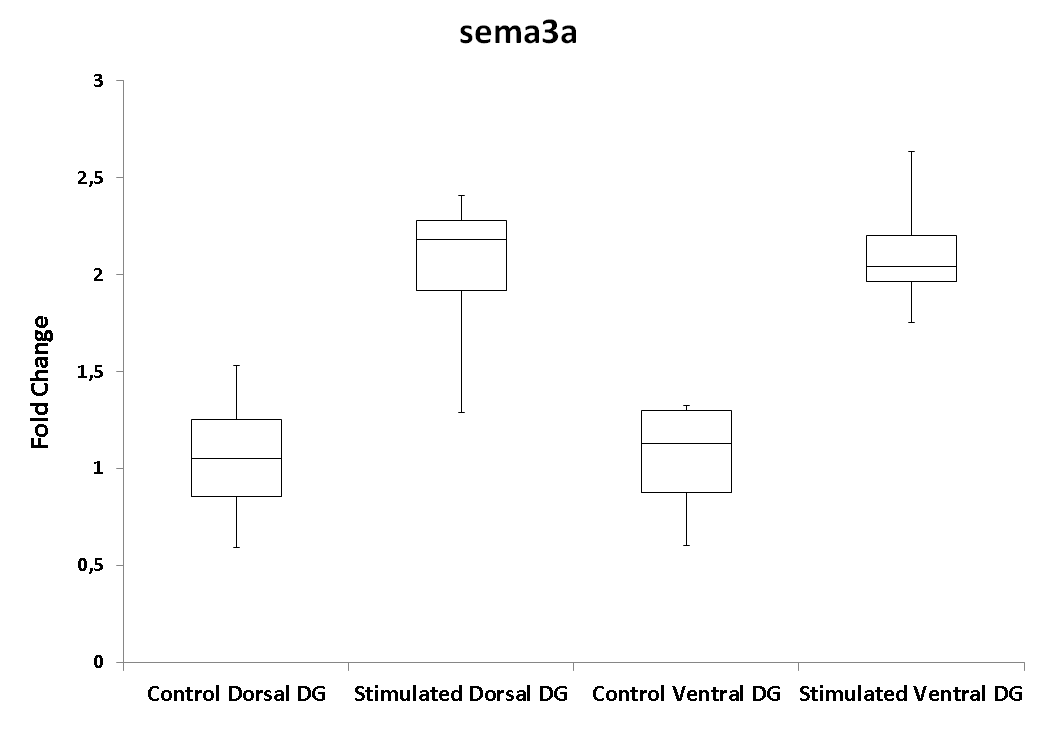


**Supplementary Figure 1.7** – Box plots of RNASeq and Real-Time PCR expression data for the *sema3a* gene. **A** – RNASeq normalized count data from DESEQ2. Statistical data from DESEQ2: Control vs Stimulated Dorsal DG p-value = 0.005; Control vs Stimulated Ventral DG p-value = 0.135. **B** – Real-Time PCR relative quantification data. T-Test: Control vs Stimulated Dorsal DG p-value = 0.023; Control vs Stimulated Ventral DG p-value = 0.005.


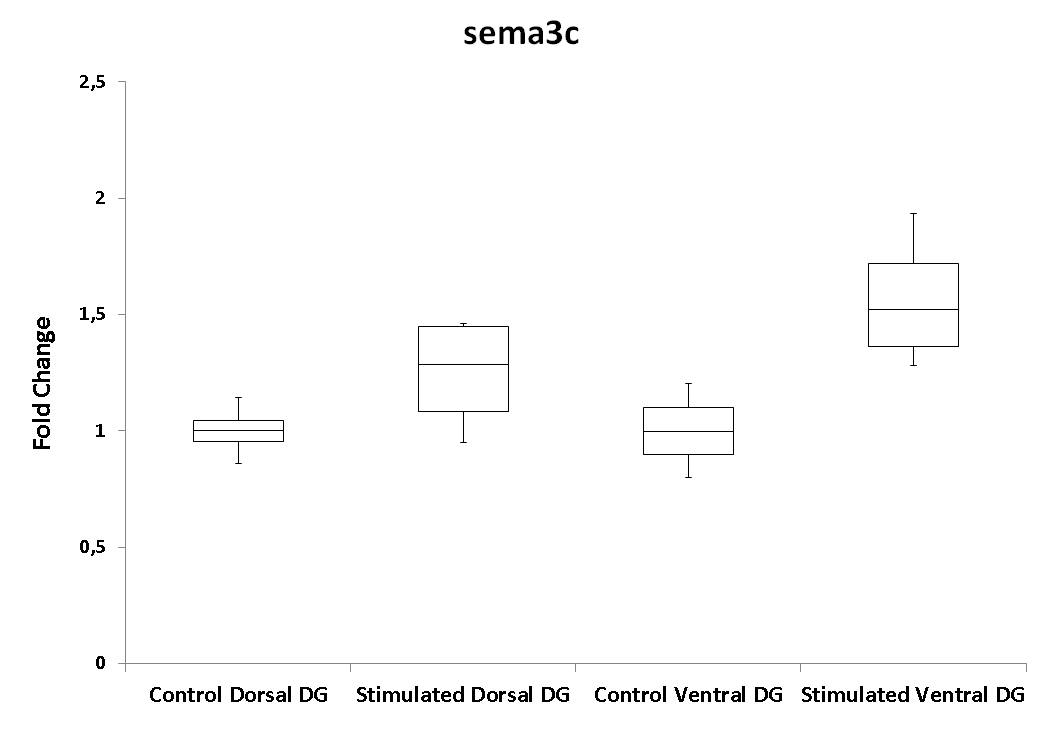


**A**

**B**


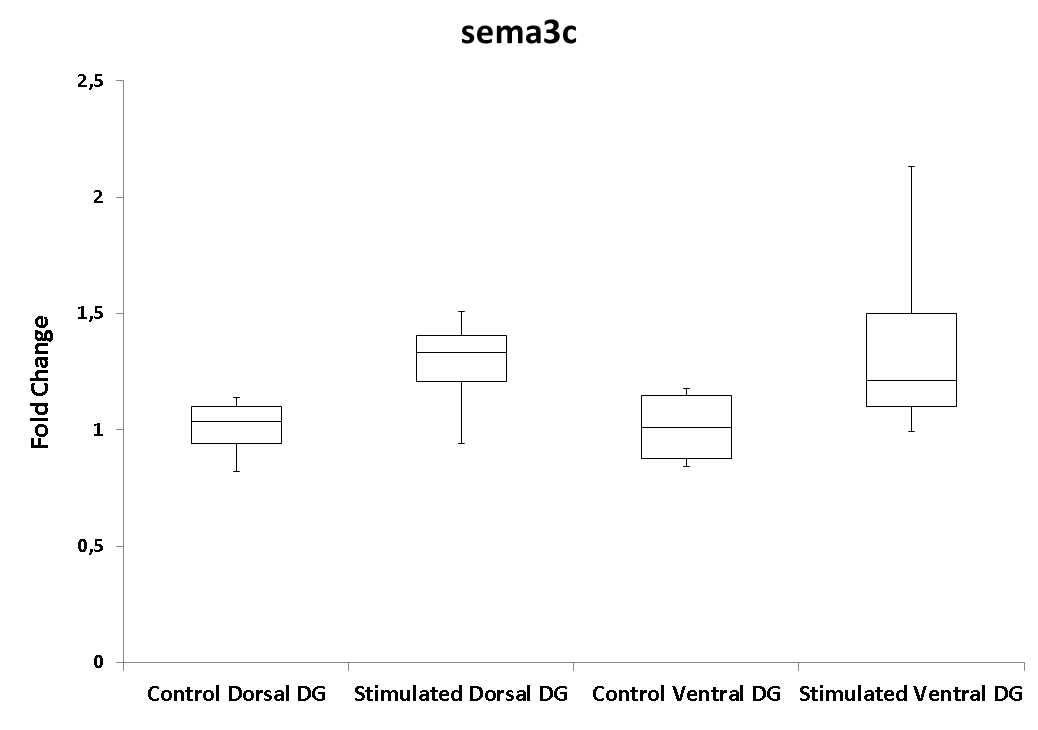


**Supplementary Figure 1.8** – Box plots of RNASeq and Real-Time PCR expression data for the *sema3c* gene. **A** – RNASeq normalized count data from DESEQ2. Statistical data from DESEQ2: Control vs Stimulated Dorsal DG p-value = 0.192; Control vs Stimulated Ventral DG p-value = 0.001. **B** – Real-Time PCR relative quantification data. T-Test: Control vs Stimulated Dorsal DG p-value = 0.099; Control vs Stimulated Ventral DG p-value = 0.211.


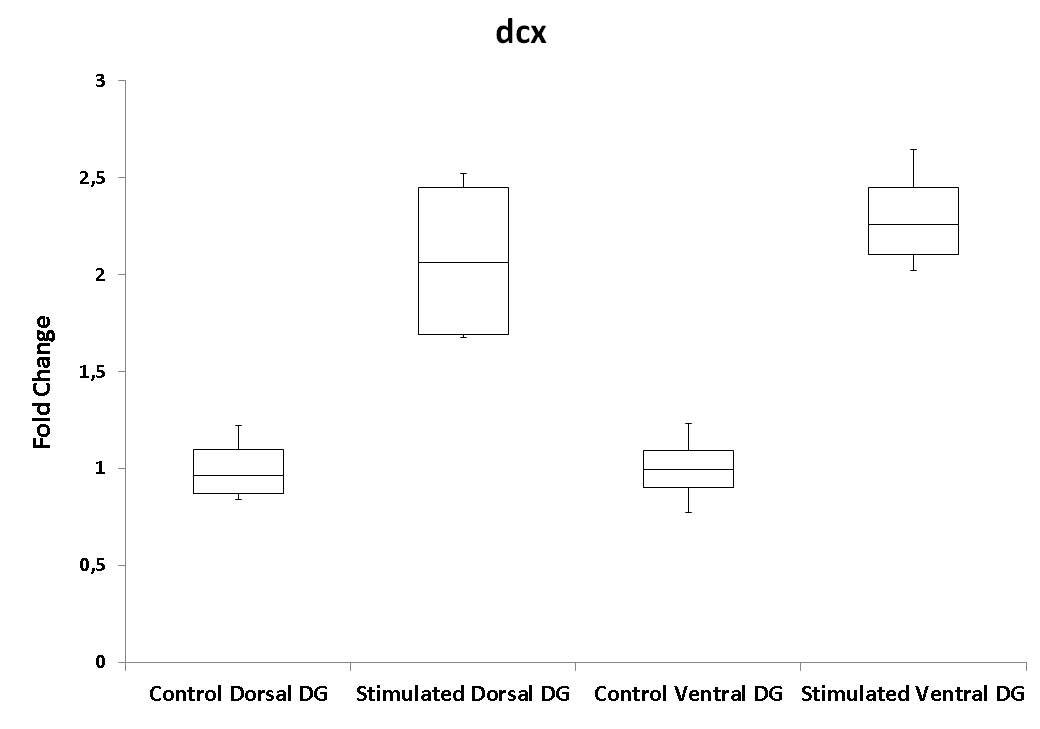


**A**

**B**


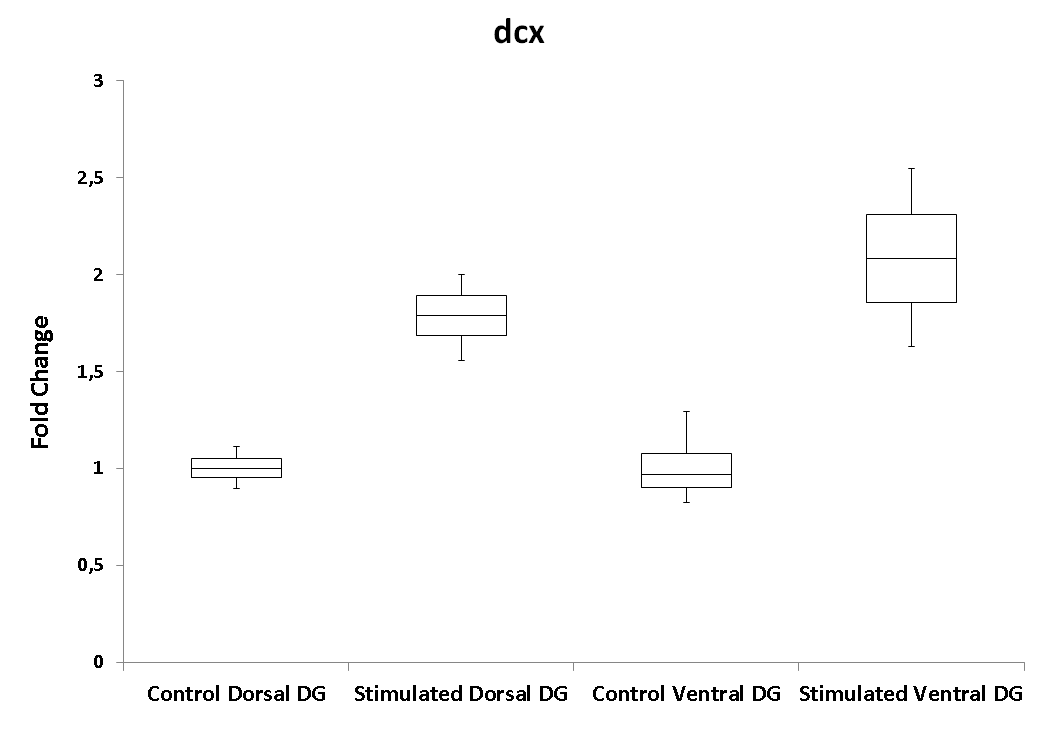


**Supplementary Figure 1.9** – Box plots of RNASeq and Real-Time PCR expression data for the *dcx* gene. **A** – RNASeq normalized count data from DESEQ2. Statistical data from DESEQ2: Control vs Stimulated Dorsal DG p-value < 0.0001; Control vs Stimulated Ventral DG p-value < 0.0001. **B** – Real-Time PCR relative quantification data. T-Test: Control vs Stimulated Dorsal DG p-value = 0.0002; Control vs Stimulated Ventral DG p-value = 0.002.


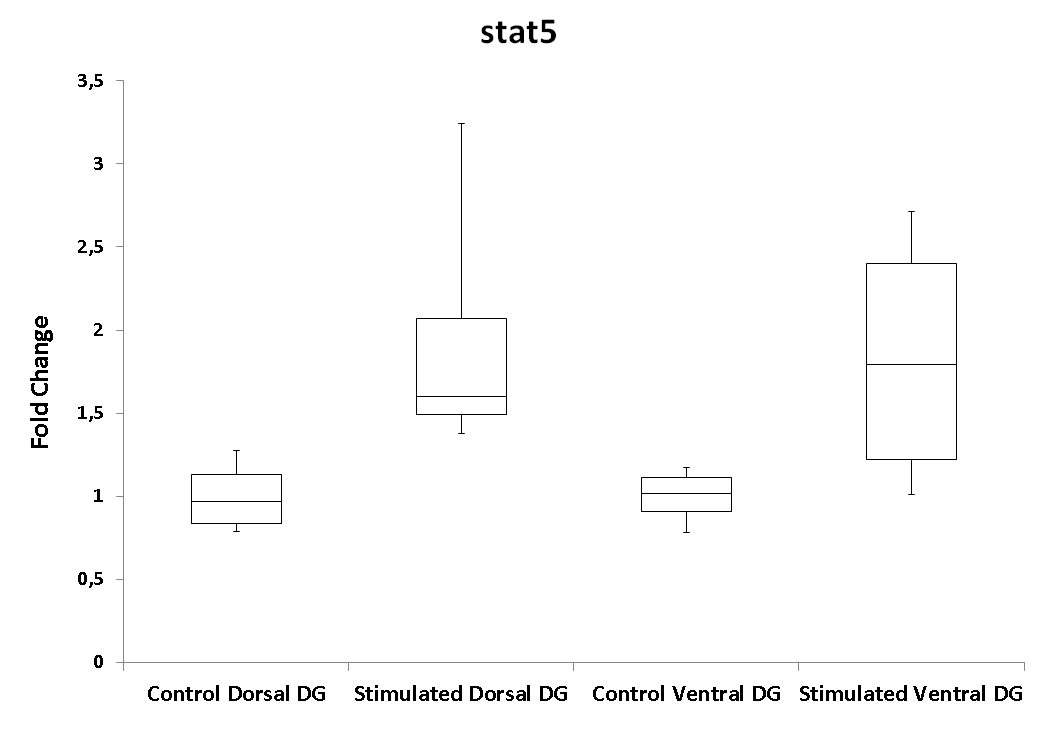


**B**

**A**

**B**


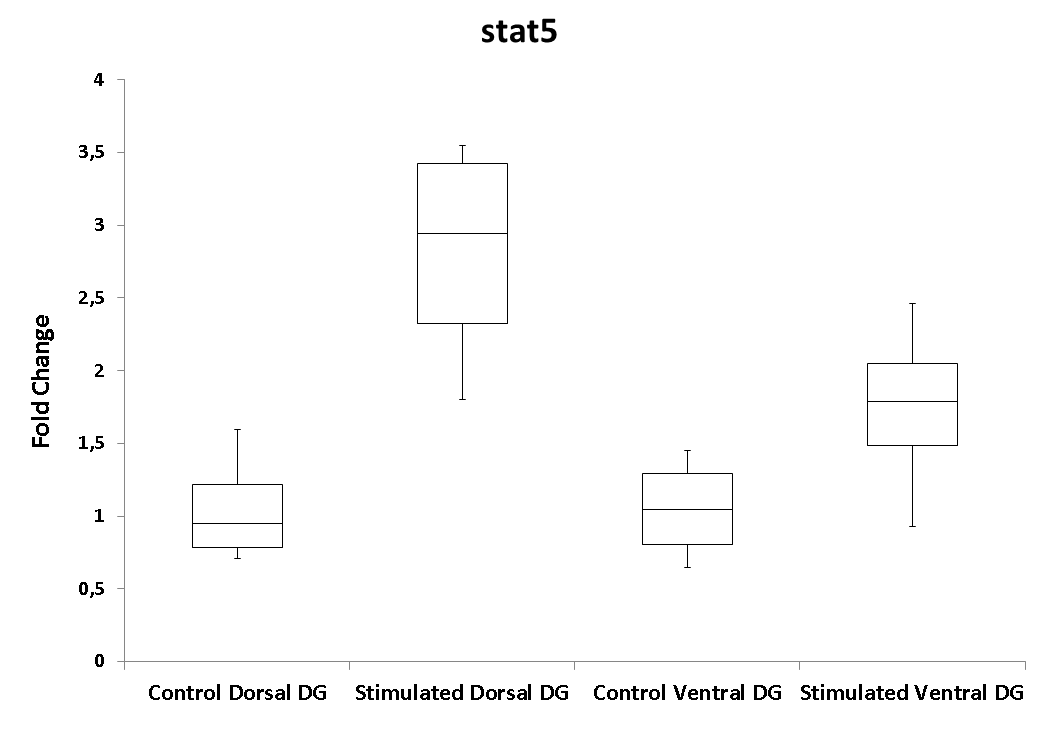


**Supplementary Figure 1.11** – Box plots of RNASeq and Real-Time PCR expression data for the *stat5* gene. **A** – RNASeq normalized count data from DESEQ2. Statistical data from DESEQ2: Control vs Stimulated Dorsal DG p-value = 0.024; Control vs Stimulated Ventral DG p-value = 0.066. **B** – Real-Time PCR relative quantification data. T-Test: Control vs Stimulated Dorsal DG p-value = 0.008; Control vs Stimulated Ventral DG p-value = 0.107.


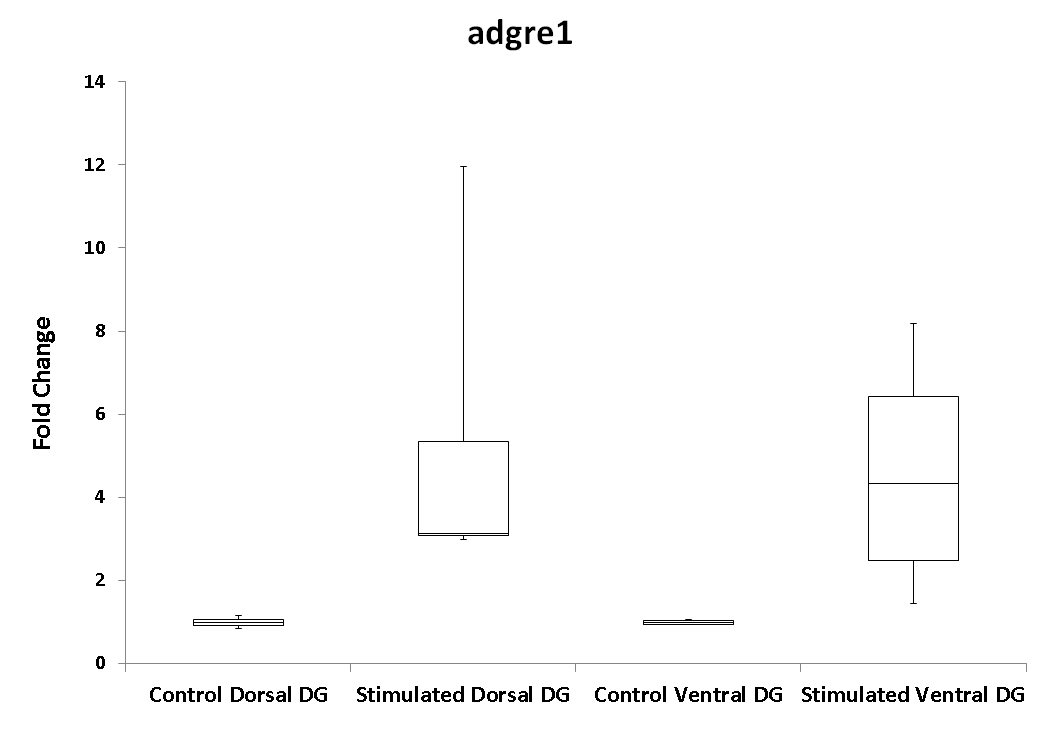


**A**

**B**


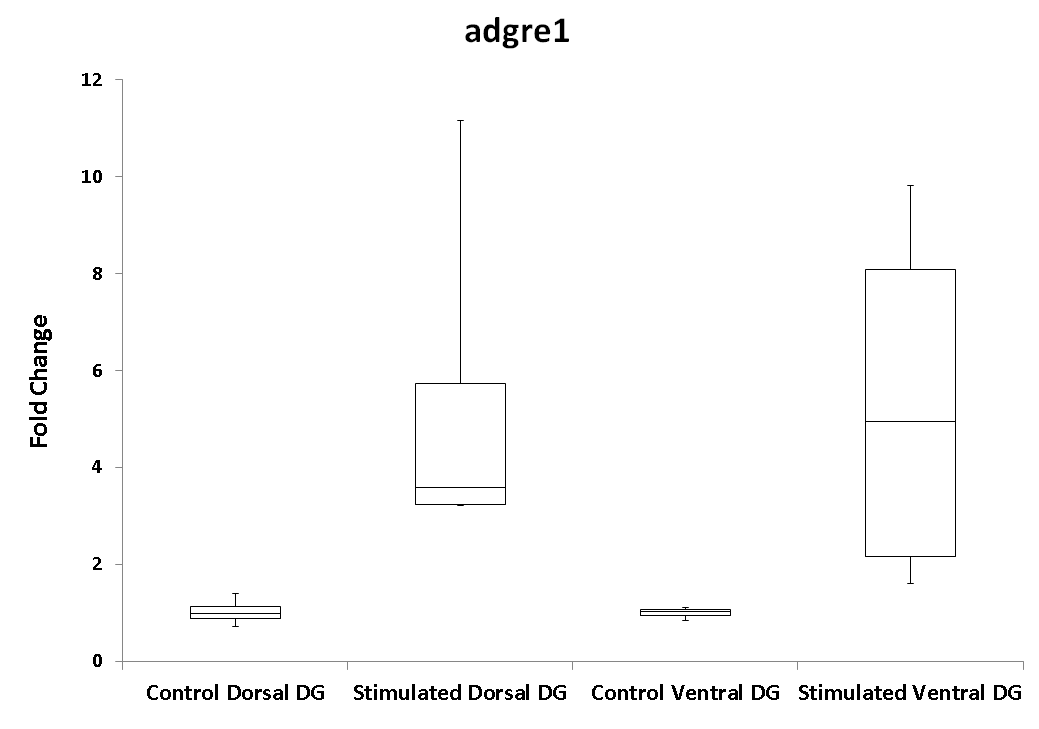


**Supplementary Figure 1.12** – Box plots of RNASeq and Real-Time PCR expression data for the *adgre1* gene. **A** – RNASeq normalized count data from DESEQ2. Statistical data from DESEQ2: Control vs Stimulated Dorsal DG p-value < 0.0001; Control vs Stimulated Ventral DG p-value = 0.0006. **B** – Real-Time PCR relative quantification data. T-Test: Control vs Stimulated Dorsal DG p-value = 0.065; Control vs Stimulated Ventral DG p-value = 0.0732.


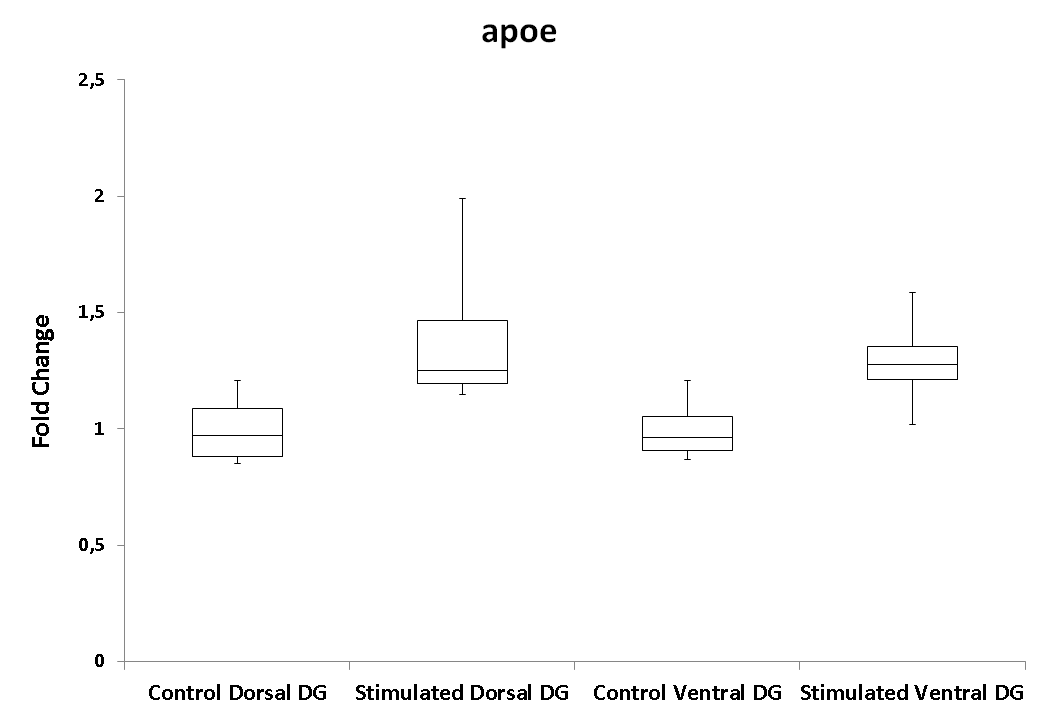


**A**

**B**


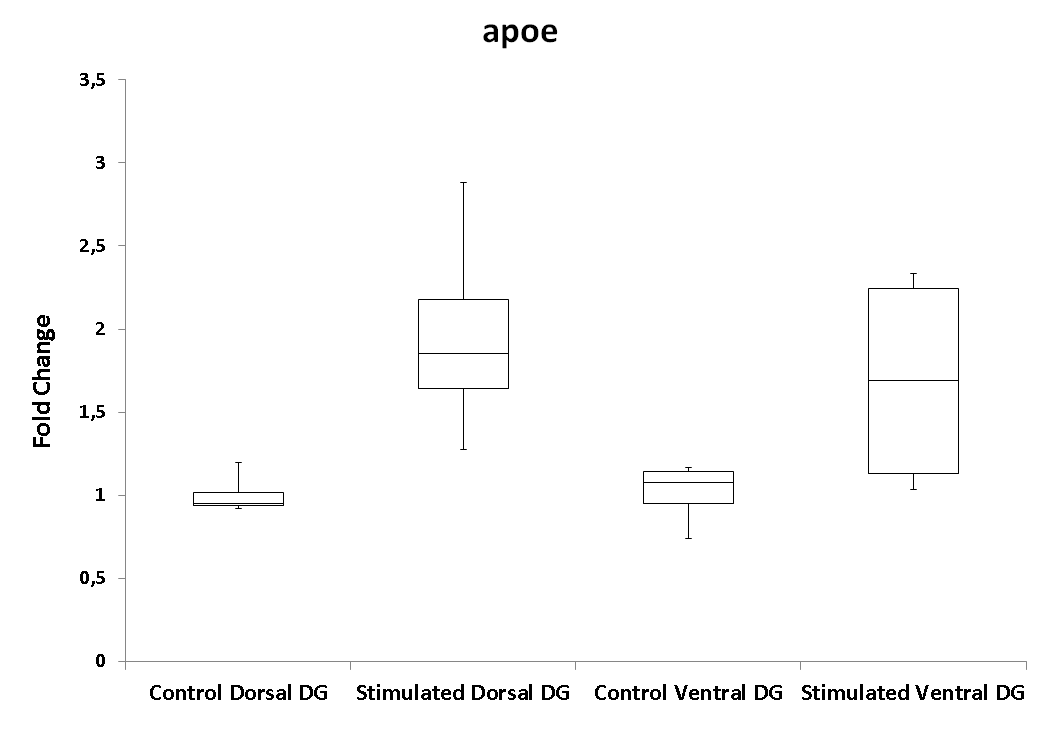


**Supplementary Figure 1.13** – Box plots of RNASeq and Real-Time PCR expression data for the *apoe* gene. **A** – RNASeq normalized count data from DESEQ2. Statistical data from DESEQ2: Control vs Stimulated Dorsal DG p-value = 0.033; Control vs Stimulated Ventral DG p-value = 0.180. **B** – Real-Time PCR relative quantification data. T-Test: Control vs Stimulated Dorsal DG p-value = 0.031; Control vs Stimulated Ventral DG p-value = 0.106.


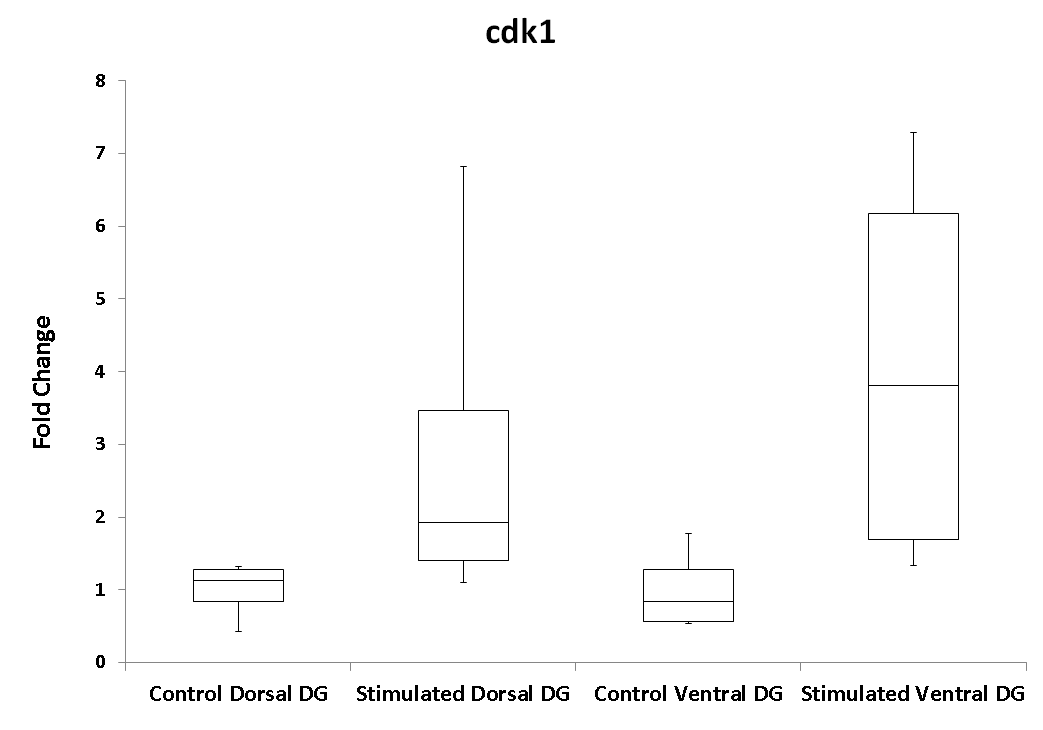


**A**

**B**


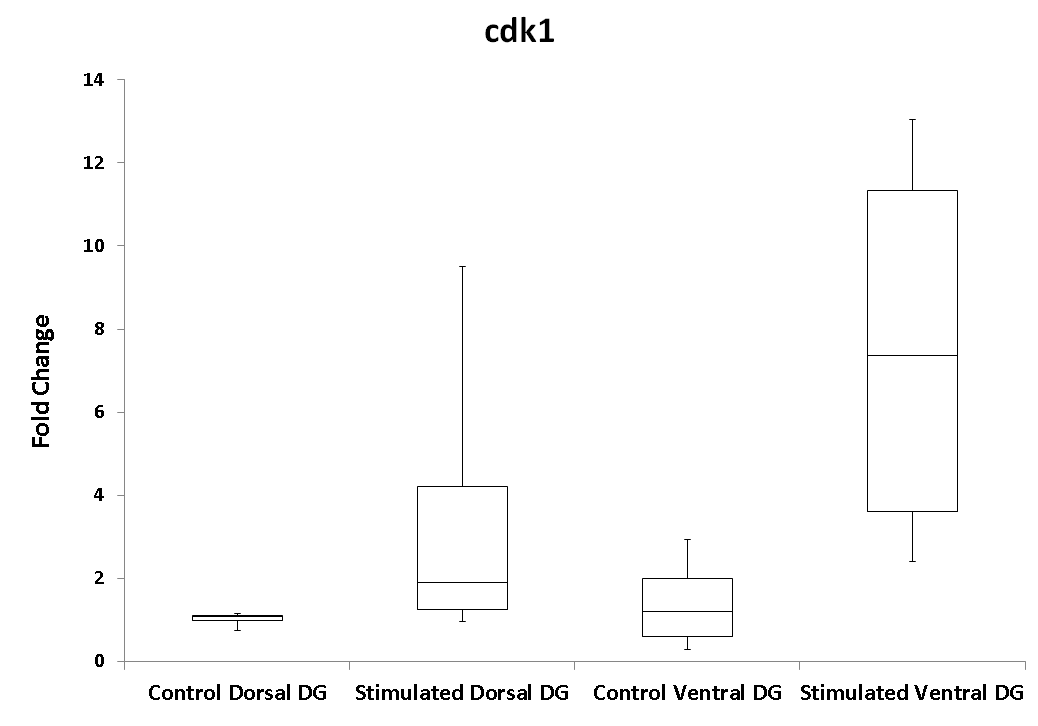


**Supplementary Figure 1.14** – Box plots of RNASeq and Real-Time PCR expression data for the *cdk1* gene. **A** – RNASeq normalized count data from DESEQ2. Statistical data from DESEQ2: Control vs Stimulated Dorsal DG p-value = 0.069; Control vs Stimulated Ventral DG p-value = 0.014. **B** – Real-Time PCR relative quantification data. T-Test: Control vs Stimulated Dorsal DG p-value = 0.25; Control vs Stimulated Ventral DG p-value = 0.058.


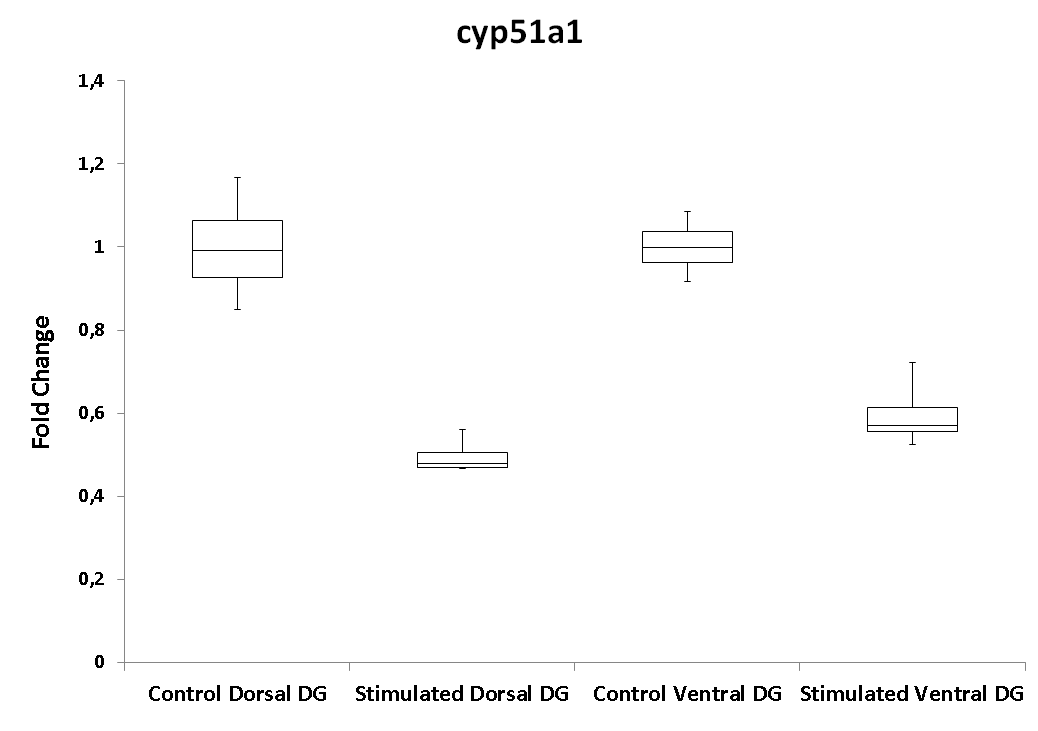


**A**

**B**


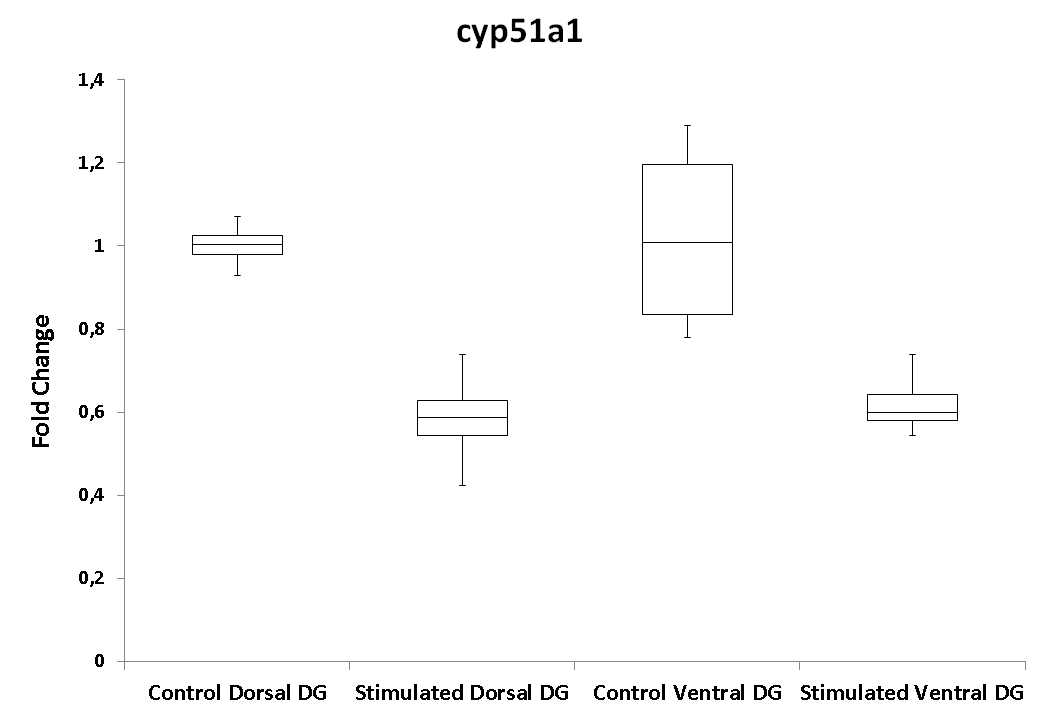


**Supplementary Figure 1.15** – Box plots of RNASeq and Real-Time PCR expression data for the *cyp51a1* gene. **A** – RNASeq normalized count data from DESEQ2. Statistical data from DESEQ2: Control vs Stimulated Dorsal DG p-value < 0.0001; Control vs Stimulated Ventral DG p-value < 0.0001. **B** – Real-Time PCR relative quantification data. T-Test: Control vs Stimulated Dorsal DG p-value = 0.001; Control vs Stimulated Ventral DG p-value = 0.021.


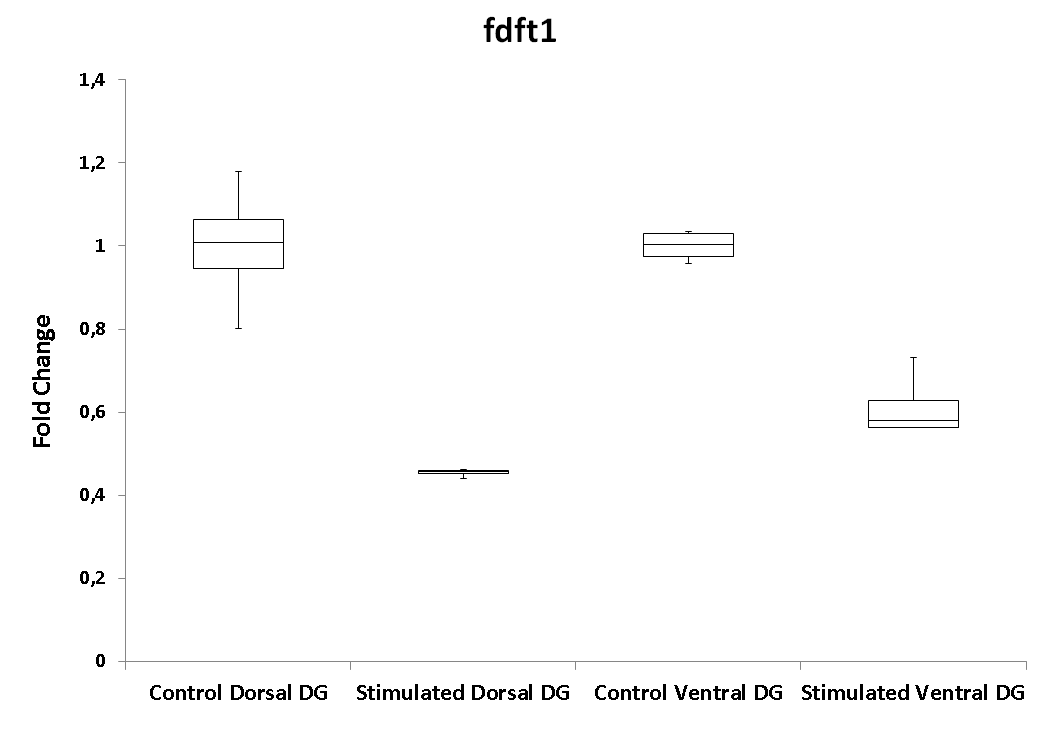


**A**

**B**


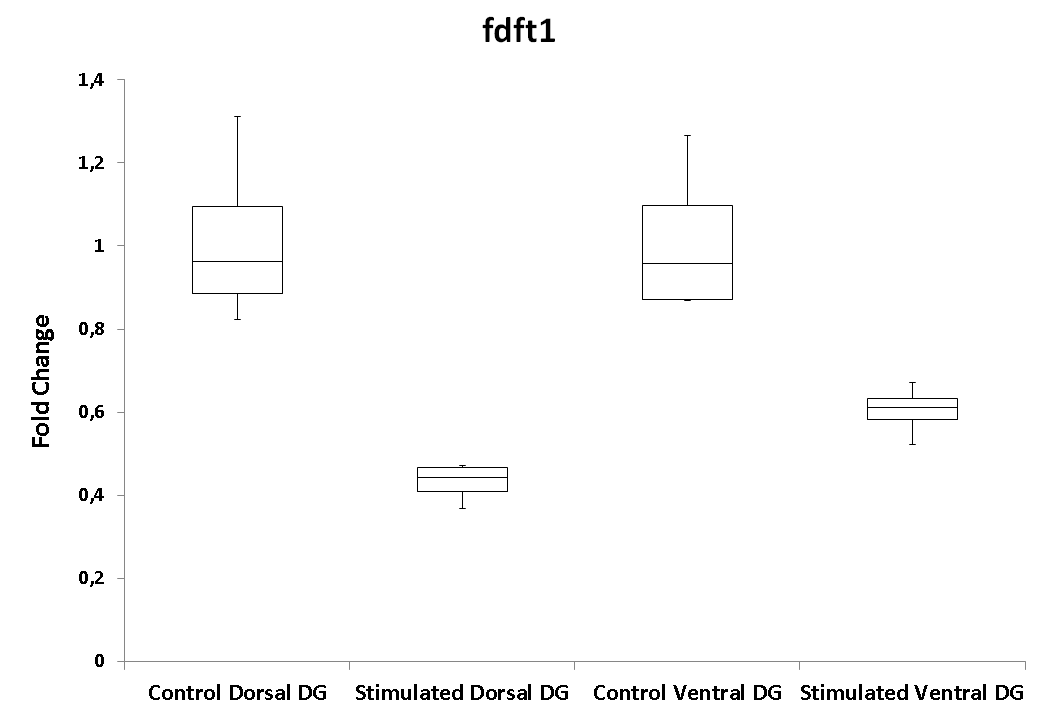


**Supplementary Figure 1.16** – Box plots of RNASeq and Real-Time PCR expression data for the *fdft1* gene. **A** – RNASeq normalized count data from DESEQ2. Statistical data from DESEQ2: Control vs Stimulated Dorsal DG p-value < 0.0001; Control vs Stimulated Ventral DG p-value < 0.0001. **B** – Real-Time PCR relative quantification data. T-Test: Control vs Stimulated Dorsal DG p-value = 0.0017; Control vs Stimulated Ventral DG p-value = 0.0061.


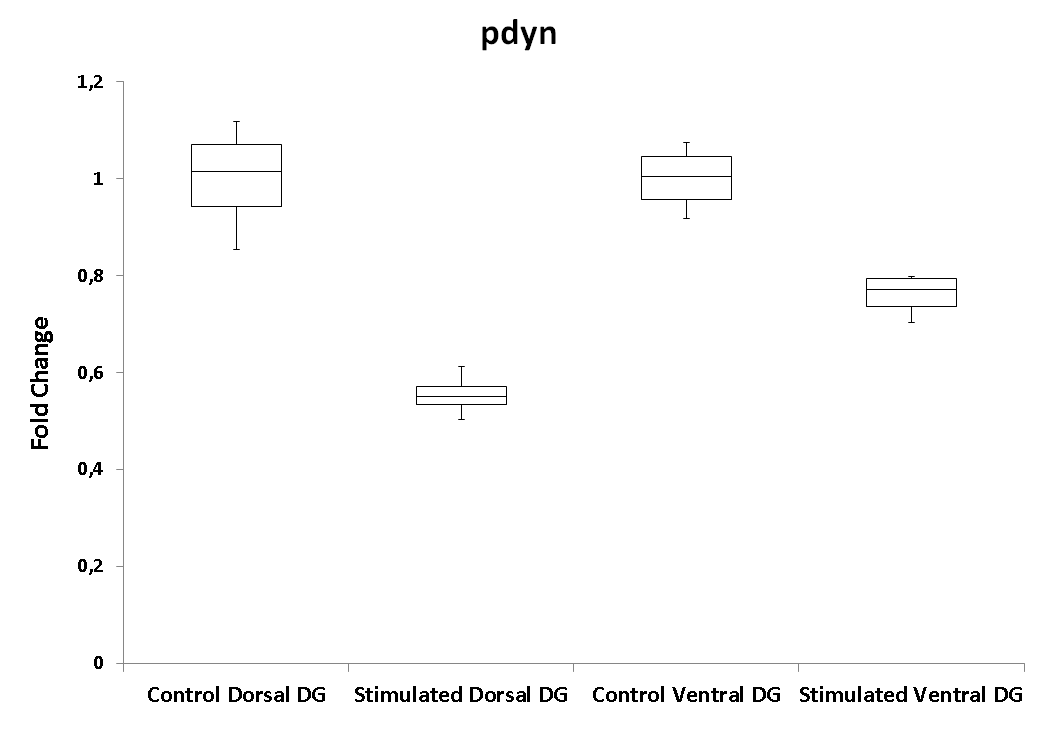


**A**

**B**


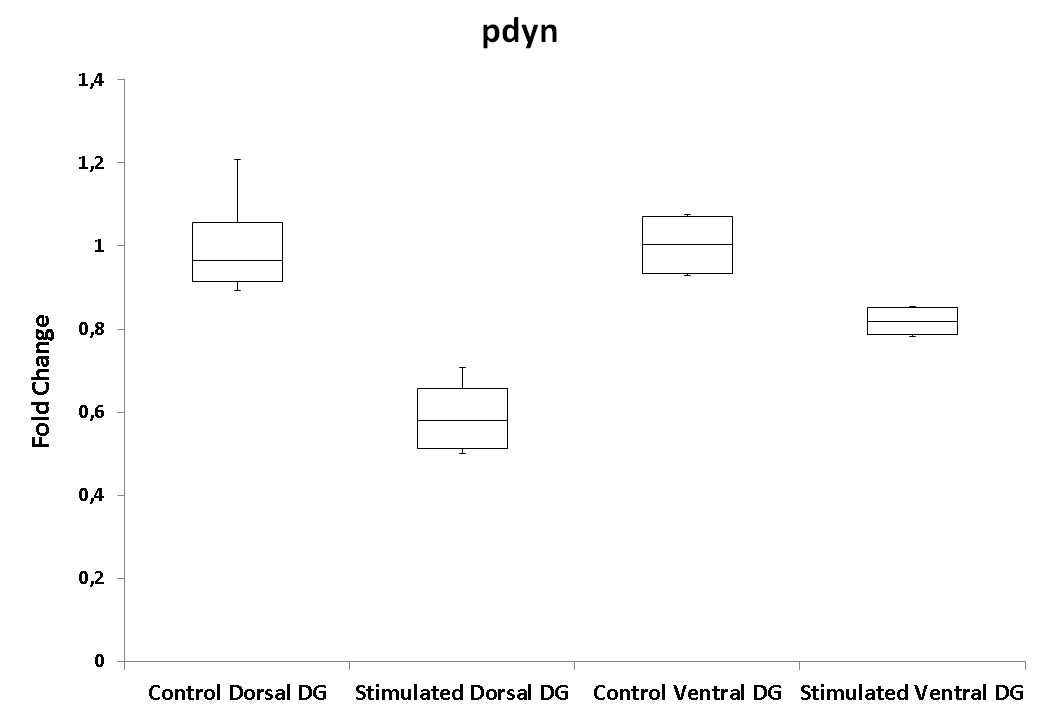


**Supplementary Figure 1.17** – Box plots of RNASeq and Real-Time PCR expression data for the *pdyn* gene. **A** – RNASeq normalized count data from DESEQ2. Statistical data from DESEQ2: Control vs Stimulated Dorsal DG p-value < 0.0001; Control vs Stimulated Ventral DG p-value = 0.0002. **B** – Real-Time PCR relative quantification data. T-Test: Control vs Stimulated Dorsal DG p-value = 0.003; Control vs Stimulated Ventral DG p-value = 0.0065.


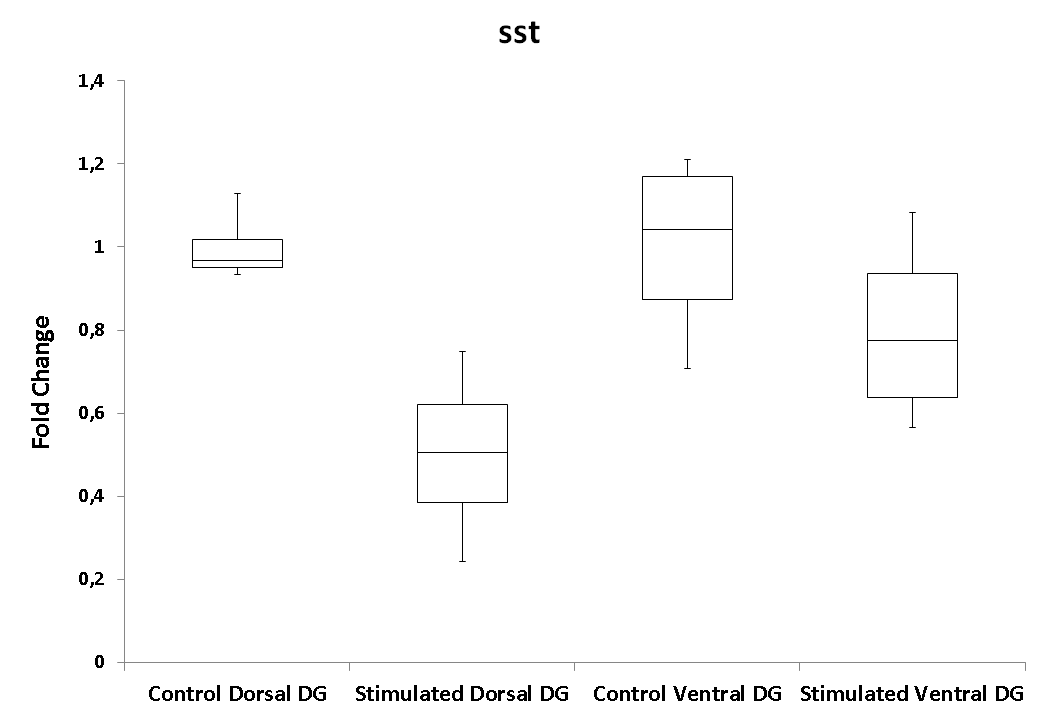


**A**

**B**


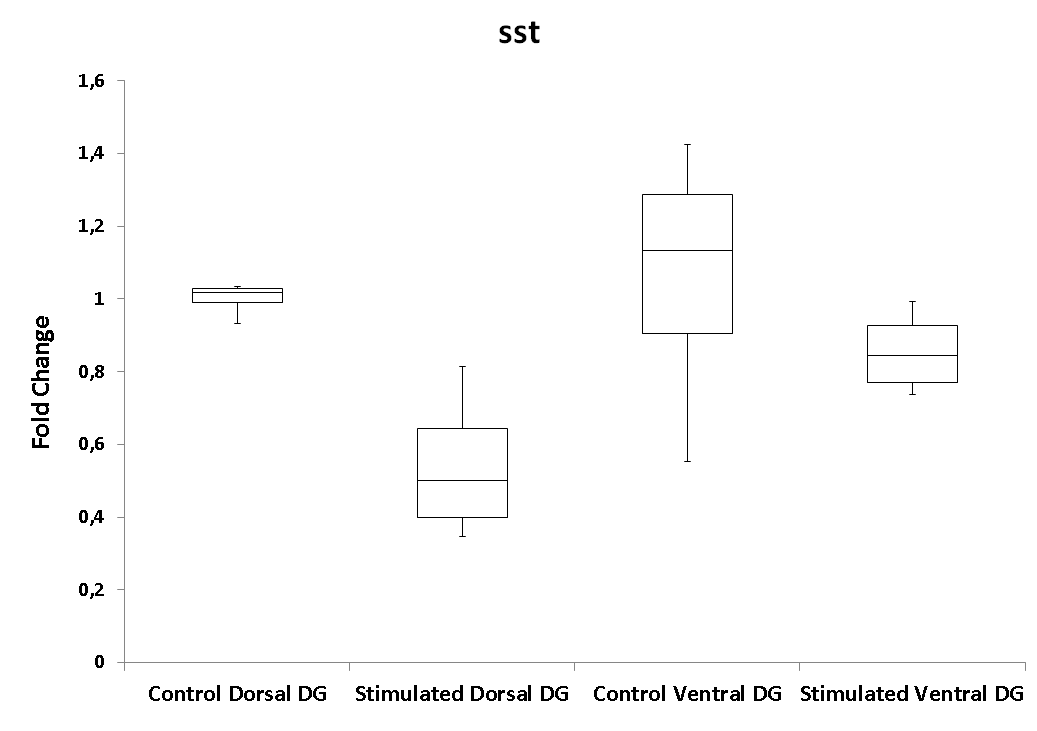


**Supplementary Figure 1.18** – Box plots of RNASeq and Real-Time PCR expression data for the *sst* gene. **A** – RNASeq normalized count data from DESEQ2. Statistical data from DESEQ2: Control vs Stimulated Dorsal DG p-value = 0.002; Control vs Stimulated Ventral DG p-value = 0.536. **B** – Real-Time PCR relative quantification data. T-Test: Control vs Stimulated Dorsal DG p-value = 0.005; Control vs Stimulated Ventral DG p-value = 0.336.


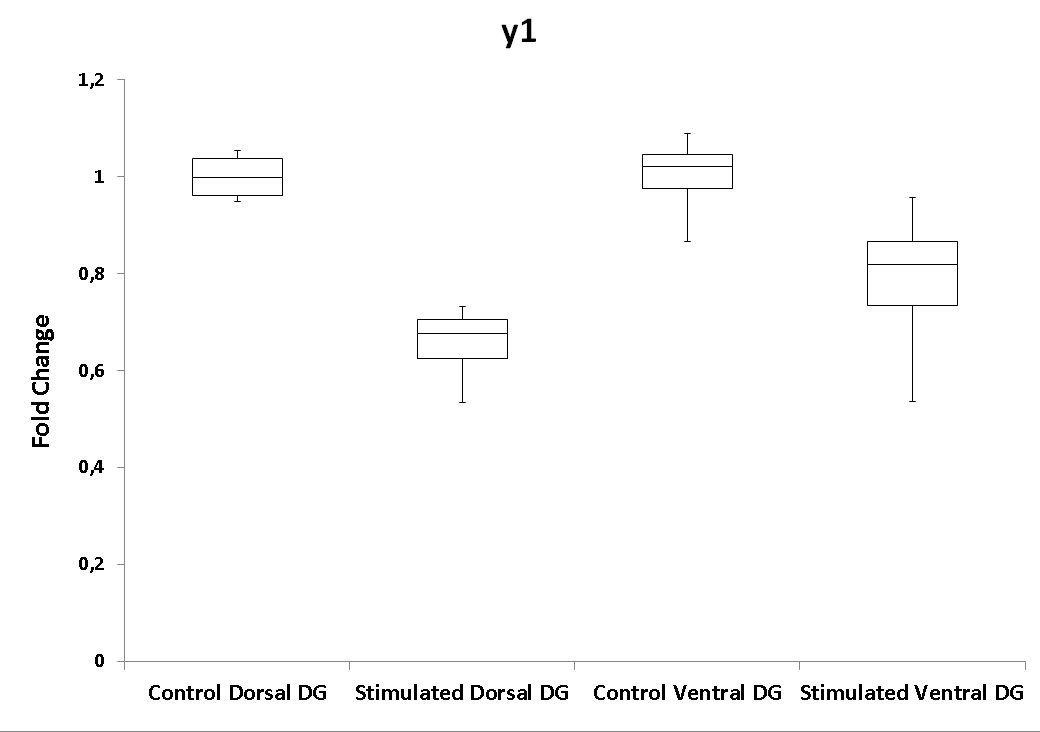


**A**

**B**


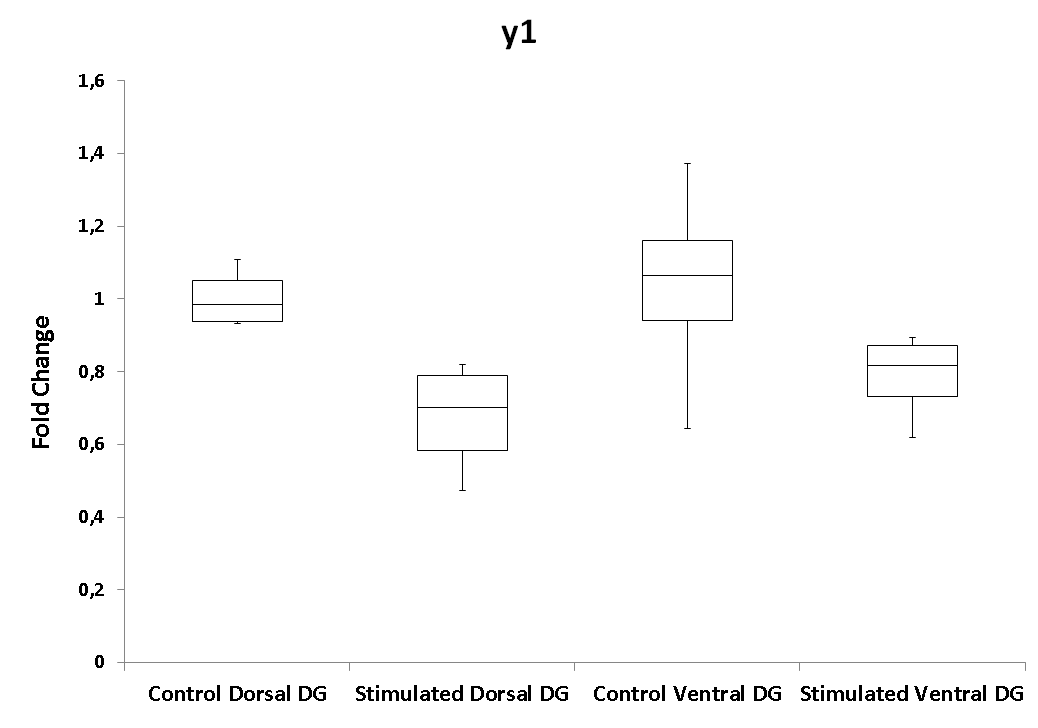


**Supplementary Figure 1.19** – Box plots of RNASeq and Real-Time PCR expression data for the *y1* gene. **A** – RNASeq normalized count data from DESEQ2. Statistical data from DESEQ2: Control vs Stimulated Dorsal DG p-value = 0.0003; Control vs Stimulated Ventral DG p-value = 0.085. **B** – Real-Time PCR relative quantification data. T-Test: Control vs Stimulated Dorsal DG p-value = 0.011; Control vs Stimulated Ventral DG p-value = 0.174.
